# Production of *p*-cresol by Decarboxylation of *p*-HPA by All Five Lineages of *Clostridioides difficile* Provides a Growth Advantage

**DOI:** 10.3389/fcimb.2021.757599

**Published:** 2021-10-29

**Authors:** Mark A. Harrison, Harparkash Kaur, Brendan W. Wren, Lisa F. Dawson

**Affiliations:** ^1^ Department of Infection Biology, London School of Hygiene and Tropical Medicine, London, United Kingdom; ^2^ Department of Clinical Research, London School of Hygiene and Tropical Medicine, London, United Kingdom

**Keywords:** *Clostridioides difficile*, *para*-cresol, *p*-cresol, *p*-HPA, virulence factors, infection pathogenesis, dysbiosis, microbiome

## Abstract

*Clostridioides difficile* is the leading cause of antibiotic-associated diarrhea and is capable of causing severe symptoms, such as pseudomembranous colitis and toxic megacolon. An unusual feature of *C. difficile* is the distinctive production of high levels of the antimicrobial compound *para*-cresol. *p*-Cresol production provides *C. difficile* with a competitive colonization advantage over gut commensal species, in particular, Gram-negative species. *p*-Cresol is produced by the conversion of *para*-hydroxyphenylacetic acid (*p*-HPA) *via* the actions of HpdBCA decarboxylase coded by the *hpdBCA* operon. Host cells and certain bacterial species produce *p*-HPA; however, the effects of *p*-HPA on the viability of *C. difficile* and other gut microbiota are unknown. Here we show that representative strains from all five *C. difficile* clades are able to produce *p*-cresol by two distinct mechanisms: (i) *via* fermentation of *p*-tyrosine and (ii) *via* uptake and turnover of exogenous *p*-HPA. We observed strain-specific differences in *p*-cresol production, resulting from differential efficiency of *p-*tyrosine fermentation; representatives of clade 3 (CD305) and clade 5 (M120) produced the highest levels of *p*-cresol *via* tyrosine metabolism, whereas the toxin A-/B+ isolate from clade 4 (M68) produced the lowest level of *p*-cresol. All five lineages share at least 97.3% homology across the *hpdBCA* operon, responsible for decarboxylation of *p*-HPA to *p*-cresol, suggesting that the limiting step in *p*-cresol production may result from tyrosine to *p*-HPA conversion. We identified that elevated intracellular *p*-HPA, modulated indirectly *via* CodY, controls *p*-cresol production *via* inducing the expression of HpdBCA decarboxylase ubiquitously in *C. difficile* populations. Efficient turnover of *p*-HPA is advantageous to *C. difficile* as *p*-HPA has a deleterious effect on the growth of *C. difficile* and other representative Gram-negative gut bacteria, transduced potentially by the disruption of membrane permeability and release of intracellular phosphate. This study provides insights into the importance of HpdBCA decarboxylase in *C. difficile* pathogenesis, both in terms of *p*-cresol production and detoxification of *p*-HPA, highlighting its importance to cell survival and as a highly specific therapeutic target for the inhibition of *p*-cresol production across *C. difficile* species.

## Introduction

*Clostridioides difficile* is a major nosocomial pathogen that causes significant mortality and morbidity globally, with an estimated 12,800 deaths in 2017 in the USA alone (CDC, 2019). *C. difficile* primarily affects patients who have been treated with broad spectrum antibiotics for an unrelated condition, resulting in damage to the gut microbiome and a resultant loss of colonization resistance to *C. difficile*, with patients who receive longer courses of therapy at a greater risk than those receiving short courses (Brown et al., 2020). While treatment of *C. difficile* is effective with either metronidazole, vancomycin, or fidaxomicin, a major feature of *C. difficile* infection (CDI) is the high proportion of patients (20–30%) who suffer from recurrence (often multiple recurrences) either as a result of reinfection or relapse (Vardakas et al., 2012). As a result, treatments that are highly specific for *C. difficile* which prevent recurrence are a current imperative.

*Para*-cresol (4-methylphenol) is an antimicrobial compound produced by *C. difficile* either through the fermentation of *p*-tyrosine (4-hydroxyphenylalanine) *via* the intermediate *para*-hydroxyphenylacetic acid (*p*-HPA) (Scheline, 1968; Dawson et al., 2011) or *via* the conversion of exogenous *p*-HPA to *p*-cresol (Harrison et al., 2020). The conversion of *p*-HPA to *p*-cresol occurs *via* the actions of the HpdBCA decarboxylase (Selmer and Andrei, 2001), encoded by the *hpdBCA* operon (Dawson et al., 2011). This operon is formed of three genes, each encoding a subunit of decarboxylase, with all three subunits being required for *p*-cresol production (Dawson et al., 2011). *C. difficile* is highly tolerant to *p*-cresol, with the hypervirulent R20291 [Ribotype (RT) 027] strain significantly more tolerant than the 630 strain (RT012) (Dawson et al., 2011). *p*-cresol selectively inhibits the growth of Gram-negative bacteria of the Gammaproteobacteria class, including *Escherichia coli*, *Proteus mirabillis*, and *Klebsiella oxytoca*, while Gram-positive species such as *Lactobacillus fermentum*, *Enterococcus faecium*, and *Bifidobacterium adoscelentis* are significantly more tolerant to *p*-cresol (Passmore et al., 2018). Furthermore, a *p*-cresol null mutant has a fitness defect in a mouse relapse model of CDI (Passmore et al., 2018), highlighting the importance of this pathway to *C. difficile* during infection. *p*-cresol production among the resident gut bacteria is unusual, with only three other identified bacterial species producing *p*-cresol to relatively high levels: *Blautia hydrogenotrophica*, *Olsenella uli*, and *Romboutsia lituseburensis* (Saito et al., 2018). These gut commensals produced more *p*-cresol than *C. difficile* when cultured in rich media over a period of 6 days (Saito et al., 2018); however, *C. difficile* is inefficient at metabolizing *p-*tyrosine to produce *p*-cresol in rich media (Dawson et al., 2011) and requires the addition of *p*-HPA, an intermediate in the pathway (Dawson et al., 2011; Harrison et al., 2020). Therefore, the conditions used by Saito et al. likely hindered the production of *p*-cresol by *C. difficile* (Saito et al., 2018). While we have demonstrated the importance of *p*-cresol to *C. difficile* virulence, the effect of *p*-HPA on growth and pathogenesis is unknown. *p*-HPA is produced via *p*-tyrosine metabolism by a variety of bacteria, including *Acinetobacter*, *Klebsiella*, and *Clostridium* species as well as being produced by mammalian cells, and is detected in all human tissues and biofluids (Wishart et al., 2018). We recently showed that exogenous *p*-HPA induces expression of the *hpdBCA* operon, which, in turn, induces high-level *p*-cresol production (Harrison et al., 2020). Therefore, exogenous *p*-HPA produced either by human cells, the gut microbiome, or a combination of both may provide *C. difficile* with a reservoir of *p*-HPA to enhance *p*-cresol production and thus provide *C. difficile* with a competitive advantage over a number of commensal species, which are vital for colonization resistance.

The *C. difficile* species is encompassed by five distinct clades (Griffiths et al., 2010; Stabler et al., 2012). Clade 1 contains strain 630, a ribotype RT012 strain isolated from a patient, in the 1980s, with severe pseudomembranous colitis and the first strain to be genome sequenced; however, this clade also contains a diverse range of both toxigenic and nontoxigenic strains (Wust et al., 1982; Knight et al., 2015). Clade 2 includes hypervirulent strains such as R20291 (RT027), which was responsible for significant outbreaks worldwide from 2004 (Loo et al., 2005; Warny et al., 2005) and remains a prevalent global ribotype (Tamez-Torres et al., 2017; Freeman et al., 2018; PHE, 2019; Marujo and Arvand, 2020). Clade 3 includes strain CD305 (RT023), a recently emerged hypervirulent lineage prevalent in the UK (Shaw et al., 2020). Clade 4 strains, including M68, a toxin A-negative RT017 strain (PHE, 2019), are prevalent in Asia (van den Berg et al., 2004; Kim et al., 2010; Kim et al., 2016; Jin et al., 2017). Finally, Clade 5, including strain M120 (RT078), has been commonly isolated in animals. However, more recently, RT078 caused human outbreaks in the Netherlands (Goorhuis et al., 2008) and is one of the most common community-acquired ribotype in Europe (Bauer et al., 2011; PHE, 2019). There have been suggestions of a further three clades of *C. difficile* (C-I, C-II, and C-III), with C-I identified as being able to cause CDI (Ramírez-Vargas and Rodríguez, 2020). However, these cryptic clades fall well below the average nucleotide identity analysis threshold, indicating that these strains are, in fact, likely to be another species (Knight et al., 2021).

In this study, we demonstrate that the representative strains from all five clades of *C. difficile* were found to produce high levels of *p*-cresol, with the expression of the *hpdBCA* operon induced in the presence of *p*-HPA. The production of HpdBCA decarboxylase was ubiquitous across all cells within a population of *C. difficile* in response to *p*-HPA. Exogenous *p*-HPA inhibits *C. difficile* growth and enhances sporulation, as well as inhibiting the growth of a number of representative commensal bacterial strains from the gut, especially Gram-negative species. Potentially, *p*-HPA induces perturbations in the cell membrane integrity of these bacteria through a combination of reduced pH and cell membrane disruption. These findings together demonstrate the importance of conversion of *p*-HPA to *p*-cresol and validates it as a specific therapeutic target that should be effective against all *C. difficile* strains.

## Materials and Methods

### Bacterial Strains and Growth Conditions

All bacterial strains used in this study are listed in **Table 1**. The routine growth of all species was carried out using Brain Heart Infusion (Oxoid) supplemented with 5 g L^-1^ yeast extract (Sigma) and 0.05% L-Cysteine (Sigma) (BHIS). All strains were grown in anaerobic conditions in a Modular Atmosphere Control System 500 (Don Whitney Scientific) at 37°C. All media underwent a minimum of 4 hour pre-equilibration in anaerobic conditions prior to inoculation.

**Table 1:** Strains and plasmids used in this study.

Strain or plasmid	Relevant features	Source or reference
*C. difficile* strains		
630Δ*erm*	Erythromycin-sensitive strain of 630—clade 1	(Hussain et al., 2005)
630Δ*erm* P_hpdB-CDS_-SNAP	630Δ*erm* carrying SNAP-tag reporter fused *via* a linker to *hpdB* coding sequence	This study
630Δ*erm* P*_hpdB_*-*phoZ*	630Δ*erm* knockout carrying *hpdB phoZ* transcriptional reporter	(Harrison et al., 2020)
630Δ*erm* Δ*codY*	Δ*codY* mutant of 630Δ*erm*	(Tremblay et al., 2021)
630Δ*erm* Δ*codY* P*_hpdB_*-*phoZ*	*codY* knockout carrying hpdB phoZ transcriptional reporter	This study
R20291	Representative strain—clade 2	(Stabler et al., 2012)
CD305	Representative strain—clade 3	(Stabler et al., 2012)
M68	Representative strain—clade 4	(Stabler et al., 2012)
M120	Representative strain—clade 5	(Valiente et al., 2012)
630Δ*erm* P_hpdB-CDS_-SNAP	630Δ*erm* carrying a translational fusion of the promoter and coding sequence of *hpdB* fused to a SNAP-tag	This study
Gut commensal strains		
*Escherichia coli*		Dr. Simon Baines
*Klebsiella oxytoca*		Dr. Simon Baines
*Proteus mirabillis*		Dr. Simon Baines
*Enterococcus faecium*		Dr. Simon Baines
*Lactobacillus fermentum*		Dr. Simon Baines
*Bifidobacterium adoscelentis*		Dr. Simon Baines
*E. coli* cloning strains		
*E. coli* NEB5α	General cloning	New England Biolabs
*E. coli* CA434	Conjugation donor	(Hussain et al., 2005)
Plasmids		
pMTL84151		(Heap et al., 2009)
P_hpdB-CDS_-SNAP	pMTL84151 carrying translational fusion of the promoter and coding sequence of *hpdB* joined to a SNAP-tag	This study
P*_hpdB_*-*phoZ*	pMTL84151 plasmid carrying transcriptional fusion of the native *hpdBCA* promoter to *phoZ* reporter	(Harrison et al., 2020)

### Growth Analysis

The growth of *C. difficile* strains (630Δ*erm* and 630Δ*erm* hpdC::CT) and intestinal species (*Escherichia coli, Klebsiella oxytoca, Proteus mirabillis, Bifidobacterium adoscelentis, Lactobacillus fermentum*, and *Enterococcus faecium*) was assessed for a minimum of 8 h. Each strain was grown overnight in BHIS before being diluted back to OD_590nm_ of 0.5. Thereafter, 1ml of which was added to 10 ml of BHIS supplemented with 0, 1, 2, 3, and 4 mg/ml *p*-HPA. To establish whether the toxicity of *p*-HPA was related to acidification of the media, the pH of BHIS media supplemented with *p*-HPA (0, 1, 2, 3, and 4 mg/ml) was measured using a Mettler Toledo Seveneasy pH meter. To mimic the acidity that results from *p*-HPA supplementation, the pH of BHIS was adjusted using hydrochloric acid to match the pH of media supplemented with 0, 1, 2, 3, and 4 mg/ml *p*-HPA; these correspond to pH levels of 6.6, 6.2, 5.8, and 5.4, respectively. Furthermore, following the completion of growth curves at this pH, the *C. difficile* cultures were filter-sterilized using 0.22-µm filter (Millipore), and the pH of the supernatant was tested. All growth curves were carried out in a minimum of biological triplicate with OD_590nm_ determined every hour for 8 h, with a final reading at 24 h with the exception of *C. difficile* grown in the presence of *p*-HPA, which was not read at 24 h due to *C. difficile* cultures clumping in the presence of *p*-HPA, making an accurate OD_590nm_ reading impossible.

### Sporulation Assays

Sporulation assays were carried out by growth of 630Δ*erm* and 630Δ*erm hpdC*::CT in BHIS media supplemented with 0, 1, 2, and 3 mg/ml *p*-HPA. Each strain was grown overnight in BHIS and then back-diluted to OD_590nm_ of 0.5, 1 ml of which was added to 10 ml of BHIS supplemented with 0, 1, 2, or 3 mg/ml *p-*HPA. These cultures were grown for 24 h. Where cultures had aggregated in the presence of *p*-HPA, these aggregations were dispersed by vigorous pipetting and vortexing. Colony-forming units per milliliter were determined for the total cell count and the spore count using 1 ml of the culture for each. Spore counts were determined from the total cell counts by heat-killing of vegetative cells at 65°C for 25 min. Dilutions were plated onto BHIS supplemented with 0.1% sodium taurocholate in technical triplicate and counted on the following day. Sporulation assays were carried out in a biological triplicate. Sporulation percentages were calculated and analyzed by Spearman rank–order correlation. Statistical analysis by regression analysis was carried out to determine significant differences between total cell counts in each condition tested. Then, *p <*0.05 was considered significantly different.

### Phosphate Release Assay

This assay was carried out as per Passmore et al. (2018) using the phosphate assay kit (Abcam). Briefly, the species to be tested were grown overnight in 10 ml BHIS, 5 ml of which was pelleted and washed twice in 5 ml Tris-buffered saline (TBS, 50 mM Tris-HCl, pH 7.5, 150 mM NaCl) before suspension in 5 ml TBS. OD_590nm_ was measured, and the suspensions were back-diluted to OD_590nm_ of 1.0. Then, 500-µl aliquots were taken and pelleted for 2 min at 17,000 x *g*, and the supernatant was removed. The pellets were then re-suspended in 500 µl of TBS with 0, 1, 2, or 3 mg/ml *p*-HPA; additionally, extra suspensions were prepared in TBS alone to determine the maximum intracellular phosphate released, which was found by boiling the sample for 15 min. After 30, 60, and 90 min, 100 µl was removed from the anaerobic chamber and pelleted, and 30 µl of the supernatant was added to 170 µl H_2_O and 30 µl ammonium molybdate and malachite green reagent in a 96-well plate. After the final 90 min, samples were added to the reagent, and the plate was incubated at room temperature for 30 min before the absorbance at OD_650nm_ was read by a Spectramax M3 plate reader. The final results were calculated by the subtraction of OD_650nm_ from the media control from each of the media conditions. The pH of the TBS with 1, 2, and 3 mg/ml *p*-HPA was measured to be pH 6.6, 4.1, and 3.8, respectively. The phosphate release assays were repeated in TBS with matched pH (to the *p*-HPA samples, using hydrochloric acid). All assays were performed in technical duplicate and biological triplicate. Statistical analysis was undertaken by ANOVA using GraphPad Prism 8 software to compare phosphate release in each condition to the TBS control. Then, *p <*0.05 was considered significantly different.

### RNA Extraction and qRT-PCR

Total RNA was isolated from all five representative *C. difficile* strains grown in BHIS media up to OD_590nm_ of 0.6 to 0.7; RNA protect was added in a 1:1 ratio and incubated at 37°C for 5 min. The cells were pelleted by centrifugation at 17,000 x *g* at 4°C, and the pellets were stored immediately at -80°C. All centrifugation steps were carried out at 4°C and 17,000 x *g* unless otherwise indicated. Pellets were thawed and processed using the RNAPro kit (MP biomedicals); 1 ml of RNAPro solution was used to re-suspend the pellets and was transferred to Lysis Matrix B tubes (MP Biomedicals) before undergoing ribolysis for 40 s at 6.0 m/s using a FastPrep-24 Classic instrument (MP Biomedicals). The sample was transferred to 300 µl chloroform and centrifuged for 15 min, and the upper phase was transferred to 100% ethanol and underwent DNA precipitation overnight at -20°C. The samples were then centrifuged for 5 min, washed with 70% ethanol (made with nuclease-free dH_2_O), and re-centrifuged. Finally, the pellets were resuspended in 50 µl of DEPC-treated water which was stored at -80°C until further processing. Then, 25 µl of extracted material was added to 20 U Turbo DNase I (Thermofisher), 80 U RNasin plus RNase inhibitor (Promega), 50 mM magnesium sulphate, 90 mM sodium acetate, and DEPC water up to 150 µl. The samples were incubated for 1 h at 37°C in a PCR thermocycler before the addition of a further 20 U Turbo DNase and 80 U RNasin Plus, followed by incubation for another hour at 37°C.

The DNase-treated samples underwent RNA purification using the RNeasy kit (Qiagen). The samples were added to 350 µl RLT buffer and 200 µl 100% ethanol and then applied to a RNeasy column and centrifuged for 15 s at 12,800 x *g*. Two wash steps, using 500 µl RPE wash buffer, were carried out, followed by drying of the column by centrifugation for 2 min. The RNA was eluted in 17 µl DEPC water, the column was allowed to stand for 3 min before a 1 min centrifugation, and the elution step was repeated twice to give an approximate volume of 50 µl eluted RNA. The eluted RNA was tested for quality (260:280 nm ratio) and concentration using a Denovix DS-11 FX instrument (Thermo scientific). Synthesis of cDNA was carried out with 1 µg of RNA using Superscript IV (Thermo scientific) as per the instructions of the manufacturer. qRT-PCR was carried out using the Kapa Sybr Fast kit (Roche), as per the instructions of the manufacturer, on an ABI-7500 Fast system (Applied Biosystems) using 2 µl of cDNA diluted 1:10 for *hpdC* and 1:100 for *16S rRNA* (internal control). Gene expression was normalized to 16S using the ΔΔCt method described previously (Livak and Schmittgen, 2001). 16S (*rrs*) was chosen as an internal control as it is a widely used and accepted control, having been shown to be among the most stable reference genes (Metcalf et al., 2010). We observed less than one cycle variation in the 16S control between the defined media (DM) and the DM supplemented with *p*-HPA. qRT-PCR was carried out in technical triplicate on five biological replicates. Regression analysis was carried out using Stata16 to determine if fold changes were significant for each strain in the presence of *p*-HPA compared to the BHIS control and if fold changes in the presence of *p*-HPA were significantly different.

### High-Performance Liquid Chromatography With Diode Array Detection

The five strains representing each clade of *C. difficile* (630Δ*erm*, R20291, CD305, M68, and M120) as well as 630Δ*erm* and the Δ*codY* strains were grown overnight in defined media (DM). The DM recipe used in this study was made by combining the salt, trace salt, vitamin, and iron sulphate solutions from Cartman and Minton (2010); however, we replaced the casamino acids described by Cartman and Minton (2010) with defined amino acids from the minimal media described by Karasawa et al. (1995) as well as using the glucose solution at 11 mM from Karasawa et al. (1995). The only addition to these published minimal media is the *p*-tyrosine concentration, which was increased to the maximum soluble concentration, 400 mg/l. Each strain was grown overnight in DM and then back-diluted on the following day to OD_590nm_ of 0.5, 1 ml of which was used to inoculate 10 ml of DM and DM with 2 mg/ml *p*-HPA, to answer the following questions, respectively: are all five clades equally efficient at producing *p*-cresol *via* (i) the fermentation of *p*-tyrosine and (ii) *via* uptake and turnover of exogenous *p*-HPA? Strains were grown for 8 h, with 1 ml samples taken after 4 and 8 h and at each time point the OD_590nm_ was also taken, to allow for *p*-HPA and *p*-cresol production to be normalized for growth. Samples for high-performance liquid chromatography (HPLC)-diode array detection (DAD) were filter-sterilized using 0.22 µm filters before freezing at -80°C prior to the HPLC analysis.

The filter-sterilized samples were transferred to HPLC vials and analyzed immediately by injecting onto the HPLC column. Separations were achieved by utilizing an Acclaim 120 (Thermofisher), C_18_, 5 μm (4.6 × 150 mm), with the mobile phase consisting of ammonium formate (10 mM, pH 2.7) and menthol (v/v; 40:60) at a flow rate of 2 ml/min. *p-*HPA and *p*-cresol were detected by the diode array detector (PDA; DAD 3000) set at 280 nm. Peak identity was confirmed by measuring the retention time of commercially available *p-*HPA and *p*-cresol, and determination of absorbance spectra was performed using the DAD. A calibration curve of each compound was generated by Chromeleon (Dionex software) using known amounts of the reference standards (0–100 mg/ml) dissolved in media and injected onto the column, and the amount of *p-*HPA and *p*-cresol in the samples was determined. Samples from three independent biological replicates were analyzed compared to media controls and standard curves. The limit of detection for *p*-HPA and *p*-cresol are 0.001 and 0.0005 mg/ml, respectively. The data was analyzed in GraphPad Prism 8, and statistical analysis was performed in Stata16 using linear regression analysis; *p <*0.05 was considered statistically significant.

### *phoZ* Assays

To determine if reduced *hpdBCA* promoter activity was the mechanism for reduced *p*-HPA turnover in 630Δ*erm* Δ*codY*, a *phoZ* transcriptional reporter fused to the *hpdBCA* promoter sequence was used (P*_hpdB_*-*phoZ*) (Harrison et al., 2020). The reporter plasmid was conjugated into 630Δ*erm* and 630Δ*erm* Δ*codY*. Assays were carried out exactly as per Harrison et al. (2020). Briefly, each strain was grown overnight in BHIS before back-dilution to OD_590nm_ of 0.5 and growth in 3:1 DM : BHIS for 3 h. The cultures were washed twice in fresh DM before a final resuspension in 1 ml of DM, 100 µl of which was added to 10 ml DM ±2 mg/ml *p*-HPA and grown for 4 h before 2-ml samples were taken, pelleted, and frozen before processing for *phoZ* activity as per Harrison et al. (2020). Phosphatase activity was calculated by the formula [OD_420 nm_ – (OD_550 nm_ × 1.75)] × 1,000/*t* (min) × OD_590_ nm × volume of cells (ml).

### Analysis of HpdB Expression and Localization

To investigate the expression and localization of HpdB, a translational fusion was constructed with the promoter region of *hpdBCA* and the HpdB coding sequence joined *via* a linker to a SNAP-tag carried in a pMTL84151 vector to give plasmid P_*hpdB*-CDS_-SNAP. The construction of the plasmid was carried out in a two-stage process. Firstly, Gibson cloning was used to insert the *hpdBCA* promoter and coding sequence into plasmid pMTL84151 (**Table 1**), followed by insertion of the linker and SNAP-tag into the pMTL84151 carrying the *hpdBCA* promoter and coding sequence (**Table 1**). All PCRs were carried out using Phusion (NEB) as per the instructions of the manufacturer. PCR products were run on a 1% agarose gel, the bands were cut out and then purified using the Monarch gel extraction kit (NEB), and ligation of PCR products was carried out using Hifi master mix (NEB) as per the instructions of the manufacturer. The primers used are listed in **Table 2**. The construction of the plasmid was confirmed by Sanger sequencing before electroporation into the conjugation strain of *E. coli* CA434 (Purdy et al., 2002). Conjugation was carried out using heat shock for 5 min at 52°C as per Kirk and Fagan (2016), with transconjugants selected by growth on BHIS with D-cycloserine and cefoxitin (*C. difficile* supplement, Oxoid) and thiamphenicol (15 µg/ml) (CCTm). Transconjugants were re-streaked once more onto BHIS CCTm plates to ensure plasmid retention.

**Table 2:** Oligos used in this study.

Primer name	Sequence (5’ to 3’)	Use
*hpdB* CDS F	ATGATTACGAAGATCTGAATTCGATAGGGTGTGC	Amplification of *hpdB* promoter and coding sequence
*hpdB* CDS R	GAGCTCGAATTTACACCCCTTCATACTCTGTTCTAGC	Amplification of *hpdB* promoter and coding sequence
84151 *hpdB* + CDS F	AGGGGTGTAAATTCGAGCTCGGTACCCG	Amplification of pMTL84151 to clone *hpdB* promoter and coding sequence
84151 *hpdB* + CDS R	ATTCAGATCTTCGTAATCATGGTCATATGGATACAG	Amplification of pMTL84151 to clone *hpdB* promoter and coding sequence
*hpdB* Linker Vec F	TGGGTAAATTCGAGCTCGGTACCCGGGGATCCTCT	Amplification of pMTL84151 *hpdB*
*hpdB* Linker Vec R	CACCACCAAGCACCCCTTCATACTCTGTTCTAGCAATTAC	Amplification of pMTL84151 *hpdB*
Linker-SNAP F	TGAAGGGGTGCTTGGTGGTGGAGGTTCAG	Amplification of the linker and SNAP-tag sequence from P_fdx_-SNAP
Linker-SNAP R	GAGCTCGAATTTACCCAAGTCCTGGTTTC	Amplification of the linker and SNAP-tag sequence from P_fdx_-SNAP
*hpdC* qRT-PCR F	GGATGCAACCAAAGGAATTTGT	Used for qRT-PCR of *hpdC*
*hpdC* qRT-PCR R	ACCCAGTCTTCTTTCTCTAGGC	Used for qRT-PCR of *hpdC*
*16S* qRT-PCR F	GGCAGCAGTGGGGAATATTG	Used for qRT-PCR of *16S*
*16S* qRT-PCR R	CCGTAGCCTTTCACTCCTGA	Used for qRT-PCR of *16S*

### Confocal Microscopy

To prepare the slides for microscopy, overnight cultures of 630Δ*erm* P_*hpdB*-CDS_-SNAP were back-diluted to OD_590nm_ of 0.5, and 1 ml was added to BHIS ±2 mg/ml *p*-HPA. These cultures were grown for 4 h before 500 µl was removed and added to 2.5 µl 50 nM TMR-Star and incubated anaerobically in the dark for 30 min. Following incubation, the samples were pelleted at 4,000 x *g* for 2 min and washed twice in 500 µl PBS; after the second wash, approximately 20 µl PBS was left in the tube and used to re-suspend the pellet. A 1-µl loop was used to spread the culture onto a glass slide, which was allowed to air dry for 1 min. Then, 10 µl of Vectashield with DAPI was added to the dried cells, with a coverslip placed over the top and sealed with clear nail polish. The slides were imaged under oil immersion using a Zeiss LSM-800 microscope (×100 objective). Excitation and emission used for the dyes were 358 and 463 nm for DAPI and 578 and 603 nm for TMR-Star, respectively. A minimum of three fields-of-view per slide was imaged; a total of 822 cells were imaged, with an average of 19 cells per field-of-view. The images were processed in Zeiss Zen Blue software.

### Western Blot and Mass Spectrometry

Confirmation of the fusion of the SNAP-tag to HpdB was carried out by anti-SNAP-tag western blotting and mass spectrometry (MS). Samples were prepared by an overnight culture of 630Δ*erm* P_*hpdB*-CDS_-SNAP being back-diluted to OD_590nm_ of 0.5, and 1 ml was added to BHIS ±2 mg/ml *p*-HPA and grown for 4 h. Following growth, 10 ml of the cultures was pelleted and frozen at -80°C. The pellets were resuspended in 1 ml of binding buffer (50 mM Tris-HCl, pH 8.0, 300 mM NaCl, 25 mM imidazole), transferred to a lysis matrix B tube, and then ribolysed at 6.0 m/s for 40 s. The samples were centrifuged, and the supernatant was saved. In duplicate, for each culture sample, 15 µl of the supernatant was added to 5 µl of 4X loading dye (Thermofisher), and the sample was heated at 95°C for 5 min before loading onto two separate 10% Bis-Tris gels which were run at 180 V, 400 A. One gel then underwent transfer to a nitrocellulose membrane using the IBlot system as per the instructions of the manufacturer; the other gel was saved for excision of the appropriate band for mass spectrometry. The membrane was washed in 1X phosphate-buffered saline (PBS) Tween20 (0.1%) for 5 min before undergoing blocking with a blocking buffer (5% whole milk in PBS Tween 20, 0.1%). Then, 10 ml blocking buffer, containing a 1:1,000 dilution of anti-SNAP antibody (New England Biolabs), was added to the membrane and incubated at room temperature for 1 h. The membrane was washed three times in PBS Tween20 (0.1%) for 5 min. Moreover, 10 ml PBS Tween20 (0.1%), containing a 1:10,000 dilution of IRDye CW800 goat anti-rabbit antibody (Li-cor), was added to the membrane and incubated for 1 h before three 5-min wash steps in PBS Tween20 (0.1%). The membrane was visualized and imaged on an Odyssey Li-cor instrument at the 800-nm wavelength. The HpdB–SNAP-tag band was identified at 121 kDa, which was excised from the second Bis-Tris gel and sent for mass spectrometry analysis at the Centre of Excellence for Mass Spectrometry at Kings College London. MS was done as previously (Dawson et al., 2021), with the following modifications: (1) 75 μM C18 column (50 cm length) was used rather than 75 μM C18 column (15-cm length), (2) Xcalibur software, v4.4.16.14, was used, (3) Proteome Discoverer software, v2.5, was used, and (4) Scaffold 5 software, v5.0.1, was used. The analysis of MS results was carried out in Scaffold software, v5.0.1, and compared to the Uniprot All Taxonomy database. Six peptides were identified as being from the HpdB of *C. difficile* strain 630, four with 100% identity and two with 99% identity, providing a coverage of 5.9% of the amino acid sequence of HpdB. The peptides identified by MS are listed in **Supplementary Table S1**.

## Results

### Representatives of *C. difficile* Clades 3 and 5 Produce Significantly More *p*-cresol From Tyrosine Fermentation

We have previously identified that *p*-cresol can be produced by two distinct yet interrelated pathways: (i) directly from tyrosine fermentation or (ii) alternatively from the turnover of exogenous *p*-HPA (Harrison et al., 2020). In this study, we sought to determine whether these two alternative mechanisms of *p*-cresol production are conserved in representatives of each C. *difficile* clade (Scheline, 1968; Dawson et al., 2011; Vardakas et al., 2012; CDC, 2019; Brown et al., 2020) and whether there are clade-specific differences in *p*-cresol production, which could potentially contribute to virulence.

**Figure 1:**
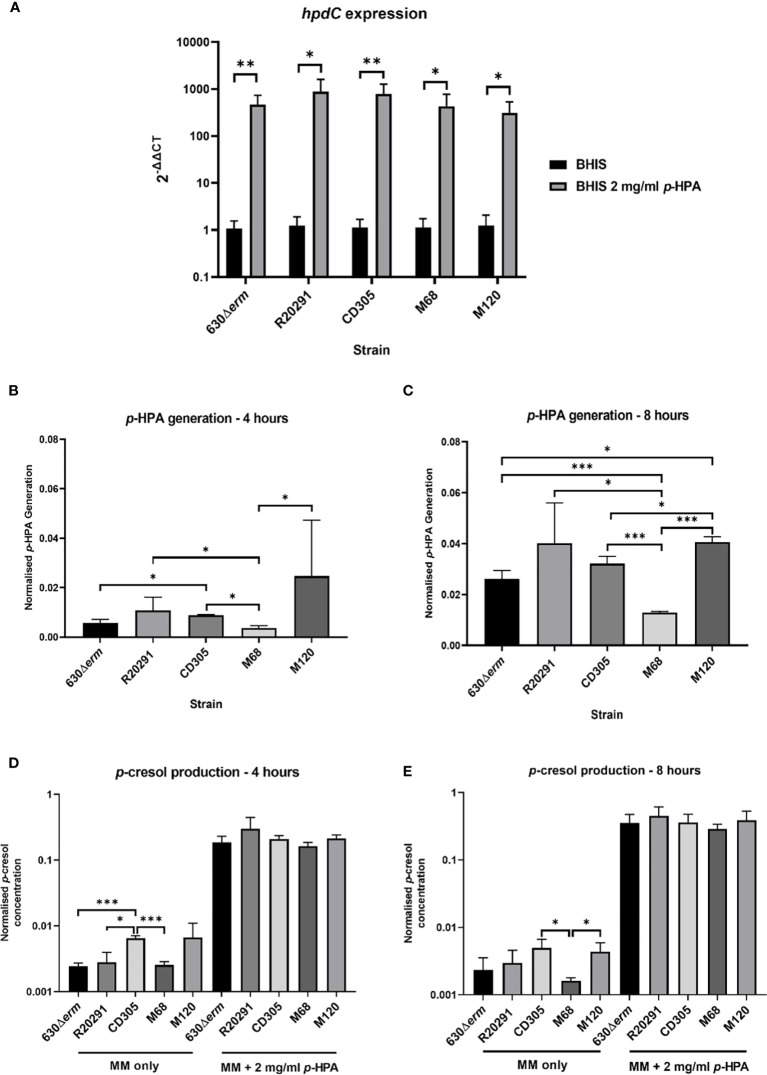
*hpdBCA* expression, *p*-HPA, and *p*-cresol production by strains representing each of the *C. difficile* clades 1–5. **(A)** qRT-PCR was used to determine whether *p*-HPA induces the expression of the *hpdBCA* operon across all five clades of *C. difficile*. Representative strains 630Δ*erm* (clade 1), R20291 (clade 2), CD305 (clade 3), M68 (clade 4), and M120 (clade 5) were grown to exponential phase (OD_590nm_ 0.6-0.7) in BHIS ±2 mg/ml *p*-HPA. The expression was normalized to 16S rRNA control and analyzed by 2^-ΔΔCt^. Data represents the mean of five biological replicates in technical triplicate, and their standard deviation with statistical analysis was carried out by linear regression. The *p*-HPA and *p*-cresol concentrations were determined by high-performance liquid chromatography on samples from each strain grown in defined media with or without 2 mg/ml exogenous *p*-HPA, with samples taken after 4 h **(B, D)** and 8 h **(C, E)**. **(B, C)** *p*-HPA generation was determined by the addition of *p*-HPA and *p*-cresol detected, and **(D, E)** *p*-cresol concentration was determined. The concentrations were normalized to growth (by dividing the concentration by the OD_590nm_ at the time the sample was taken). Data represents the mean and standard deviation of three independent replicates. Statistical analysis was carried out by linear regression and used to determine significant differences between normalized *p*-HPA generation and *p*-cresol concentrations for each strain. Statistically significant differences are indicated: *p < 0.05, **p < 0.01, ***p < 0.001.

To assess clade-dependent *p*-cresol production, we identified that the sequenced representatives of all five clades share at least 97.3% homology across the *hpdBCA* operon. We then determined the expression level of the *hpdBCA* operon controlling the production of the HpdBCA decarboxylase responsible for the turnover of *p*-HPA to *p*-cresol, alongside measuring the metabolites directly *via* HPLC. We show a significant induction of the *hpdBCA* operon in the presence of exogenous *p*-HPA, which is conserved in representative strains from all five *C. difficile* lineages: clade 1, 630Δ*erm* (RT012); clade 2, R20291 (RT027); clade 3, CD305 (RT023); clade 4, M68 (RT017); and clade 5, M120 (RT078) (**Figure 1A**). The largest fold change in the expression of the *hpdC* gene in response to *p*-HPA was observed in strains R20291 (879.9 ± 331.0) and CD305 (792.7 ± 215.3), compared to 630Δ*erm* (469.4 ± 120.7), M68 (447.7 ± 153.0), and M120 (312.3 ± 98.9) (**Figure 1A**).

Using HPLC-DAD, we were able to differentially quantify *p*-HPA and *p*-cresol production from (i) the fermentation of *p*-tyrosine (without exogenous *p*-HPA) and (ii) the uptake and turnover of exogenous *p*-HPA.

The fermentation of tyrosine in the five clades led to the identification of significant differences in *p*-HPA and *p-*cresol generation (**Figure 1**). M120, R20291, and CD305 produced the highest levels of *p*-HPA at both 4 h (137, 66.5, and 45.7 µM, respectively) and 8 h (228, 247, and 181 µM, respectively) (**Figures 1B, C**). After 8 h of normalizing for growth, M120 produced significantly more *p*-HPA than 630Δ*erm* (*p* = 0.004), CD305 (*p* = 0.018), and M68 (*p* < 0.001) (**Figure 1C**). As anticipated, *p*-cresol production tracked with *p*-HPA production from tyrosine fermentation (**Figure 1**). Strain CD305 (RT023) produced the highest level of *p*-cresol at both 4 h (17.6 µM) and 8 h (42.2 µM) (**Figures 1D, E**). After 4 h, strain CD305 produced significantly more *p*-cresol (0.0046 ± 0.0015 mg/ml) than 630Δ*erm* (0.0035 ± 0.0020 mg/ml, *p* < 0.001), R20291 (0.0025 ± 0.0017 mg/ml, *p* = 0.017), and M68 (0.0014 ± 0.0001, *p* < 0.001). After 8 h of growth, strains CD305 (0.0046 ± 0.001 mg/ml) and M120 (0.0058 ± 0.0022 mg/ml) both produced the highest levels of *p*-cresol, with strain M68 producing the least *p*-cresol (*p*<0.005) (**Figure 1E**). This highlights the strain-specific differences in *p*-cresol production, most likely as a result of the differential rate of tyrosine fermentation to *p*-HPA.

As anticipated, all representatives of clades 1–5 were able to uptake exogenous *p*-HPA and subsequently decarboxylate this to *p*-cresol, producing 2.5 to 5.1 mM *p*-cresol at 8 h. We observed an increase greater than 30-fold in *p*-cresol production in the presence of exogenous *p*-HPA compared to the *p*-cresol produced from tyrosine fermentation in all five strains (**Figure 1**). Interestingly, after normalizing for growth, no significant differences in *p*-cresol concentration were observed under *p*-HPA induction, indicating that all five strains sense, take up, and convert *p*-HPA with similar efficiency.

### The HpdBCA Decarboxylase Is Produced Ubiquitously in *C. difficile* Cells

**Figure 2:**
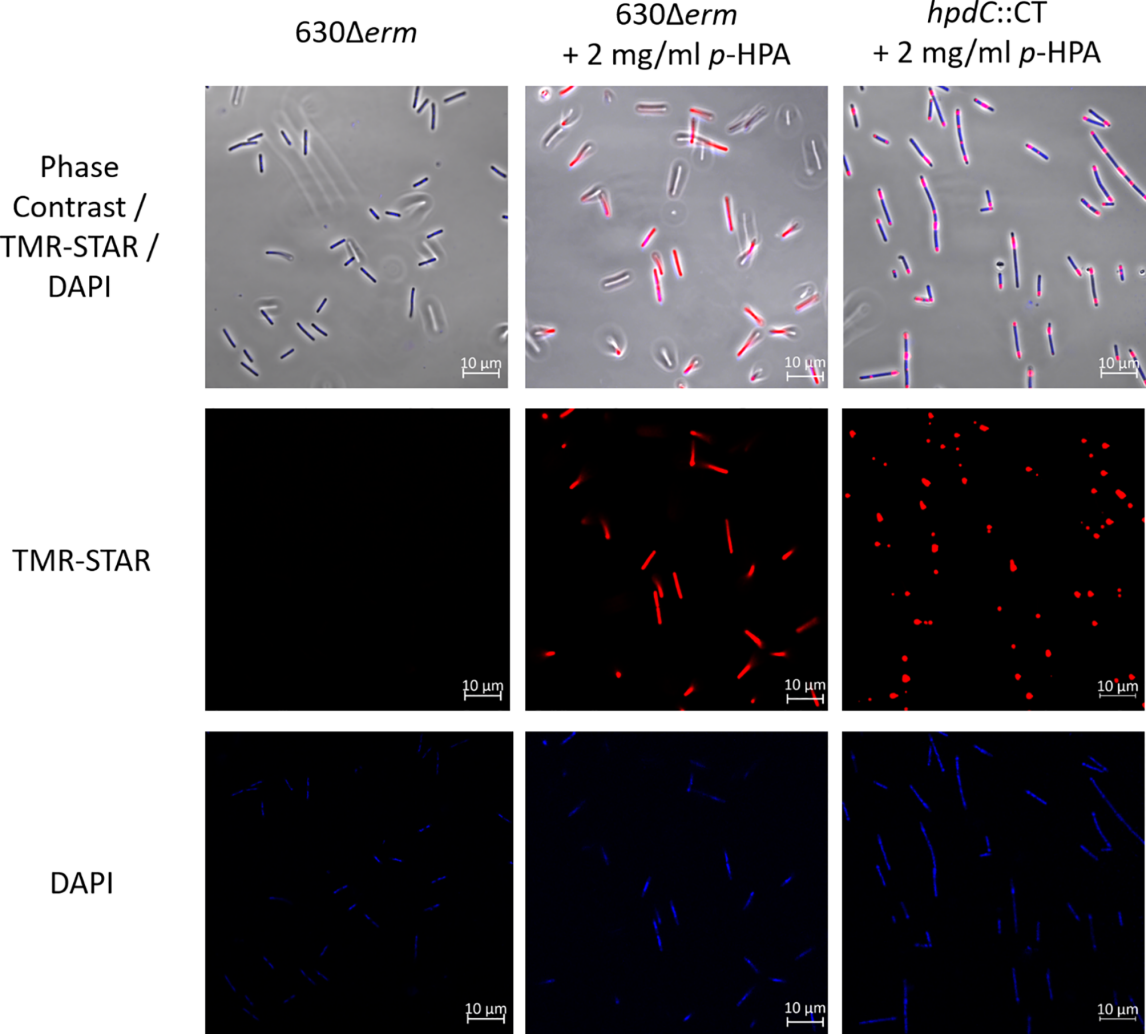
Confocal microscopy to confirm the expression and determine the localization of HpdB. A plasmid-based translational fusion of HpdB was constructed in *C. difficile* strain 630Δ*erm*, using the native *hpdBCA* promoter and *hpdB* coding sequence (without a stop codon) fused *via* a linker to the SNAP-tag to produce P_hpdB-CDS_-SNAP. The expression of HpdB linked to the SNAP-tag was confirmed by anti-SNAP western and mass spectrometry. Cultures of *C. difficile* 630Δ*erm* and *hpdC*::CT, each carrying P_hpdB-CDS_-SNAP, were grown for 4 h in BHIS ±2 mg/ml *p*-HPA, then harvested in the presence of the SNAP-tag substrate TMR-Star before staining with Vectashield DAPI, and were imaged on a Zeiss LSM880 confocal microscope at 578-nm excitation and 603-nm emission (for TMR-Star) and 358-nm excitation and 463-nm emission for DAPI. The images are representative of three independent replicates.

To investigate the global response of cells within a given *C. difficile* population to the metabolite *p*-HPA and to identify the cellular localization of the HpdBCA decarboxylase complex, a plasmid-based HpdB–SNAP-tag translational fusion was constructed using a *C. difficile*-compatible plasmid (pMTL84151) under control of the *hpdBCA* promoter. The *hpdB* coding sequence omitting the stop codon was fused via a linker to a SNAP-tag (**Table 1**). Confirmation of HpdB linked to the SNAP-tag was carried out via western blot (**Supplementary Figure S1**) and mass spectrometry, in which we identified six peptides, four with 100% identity and two with 99% identity unique to HpdB (**Supplementary Table S1**). Localization of HpdB was visualized by confocal microscopy in 630Δ*erm* and the *p*-cresol deficient mutant (*hpdC*::CT) carrying the HpdB–SNAP-tag fusion (P_hpdB-CDS_-SNAP) in the presence and absence of *p*-HPA to induce HpdBCA production. The SNAP-tag substrate TMR-Star (excitation/emission: 578/603 nm) was added to visualize the HpdB–SNAP-tag, and DAPI (excitation/emission: 358/463 nm) was used to visualize DNA within the cells. Confocal microscopy revealed that all cells grown in the presence of *p*-HPA expressed the HpdB–SNAP-tag in response to *p*-HPA, whereas those grown without *p*-HPA showed very little or no visible expression (**Figure 2**). Furthermore, the HpdB–SNAP-tag fusion was located throughout the cytoplasm of the cell in the wild-type strain (**Figure 2**); however, in contrast, the HpdB–SNAP-tag in the *hpdC* knockout strain was localized in aggregates within the cells. This suggests that the HpdBCA decarboxylase complex is formed and located throughout the cytosol in all cells within a population of *C. difficile* in response to *p*-HPA.

### Mutation of CodY Leads to Reduced *p*-HPA Turnover to *p*-Cresol

**Figure 3:**
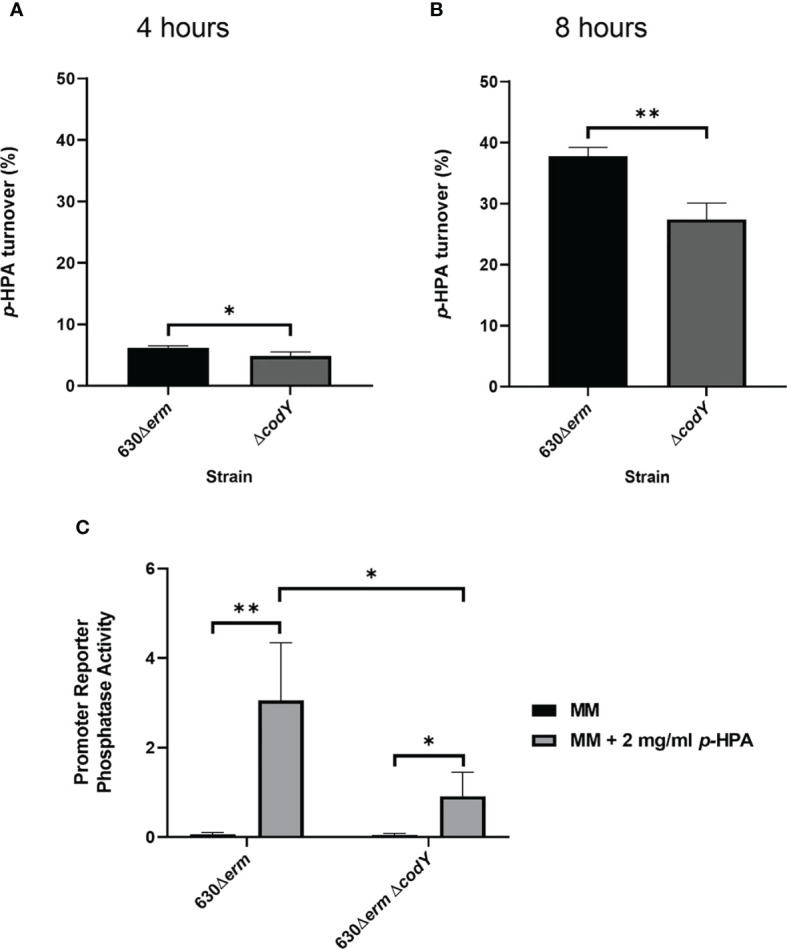
Mutation of *codY* reduces *p*-HPA conversion to *p*-cresol *via* a reduction in the expression of the *hpdBCA* operon. **(A, B)** 630Δ*erm* and 630Δ*erm* Δ*codY* were grown in defined media with 2 mg/ml *p*-HPA for 8 h, with the samples taken at 4 and 8 h for HPLC analysis of *p*-HPA and *p*-cresol concentration. The turnover percentage was calculated as (*p*-cresol/*p*-HPA + *p*-cresol) normalized to growth by OD_590 nm_. **(C)** Expression from the *hpdBCA* promoter was assessed by phosphatase activity in 630Δ*erm* and 630Δ*erm* Δ*codY* carrying P*_hpdB_*-*phoZ* grown for 4 h in DM ±2 mg/ml *p*-HPA. Phosphatase activity was calculated by the formula [OD_420 nm_ – (OD_550 nm_ x 1.75)] × 1,000/*t* (min) × OD_590_ nm × volume of cells (ml). Data represents the mean and standard deviation of three independent replicates. Statistical analysis was carried out by linear regression and used to determine significant differences between normalized *p*-HPA turnover and promoter reporter activity for each strain. *p < 0.05, **p < 0.01.

The regulation of the *hpdBCA* operon in response to exogenous *p*-HPA is not yet completely understood. Here we identify that CodY, a global transcriptional regulator that responds to the presence of branched chain amino acids (Shivers and Sonenshein, 2004) and GTP (Ratnayake-Lecamwasam et al., 2001), indirectly activates *p*-cresol production (**Figure 3**). Using a transcriptional fusion of the native *hpdBCA* promoter fused to a *phoZ* reporter (P*_hpdB_*-*phoZ*), we show a reduction of 32.9 ± 13.7% in the expression of the *hpdBCA* operon in a CodY-deficient mutant (Δ*codY*) compared to its wild-type parental strain, 630Δ*erm*, when grown in DM with exogenous *p*-HPA (2 mg/ml) (**Figure 3C**). This decrease in the expression of the *hpdBCA* operon in the *codY* mutant is translated to a decrease in the decarboxylation of *p*-HPA to *p*-cresol at both 4 and 8 h (**Figures 3A, B**). This significant deficiency in turnover of exogenous *p*-HPA to *p*-cresol was more pronounced at the later growth stage (8 h), with 27.4% (± 2.2) turnover of *p*-HPA in the *codY* mutant compared to 37.8% (± 1.2) in the wild type (*p* = 0.004). We were unable to detect a significant difference in the decarboxylation of *p*-HPA to *p*-cresol *via* tyrosine fermentation (**Supplementary Figure S2**), suggesting that CodY is involved in sensing and responding to exogenous *p*-HPA, most likely indirectly as there is no CodY binding site found in the promoter region (Dineen et al., 2010).

We show that the expression of the *hpdBCA* operon in all five clades is induced in the presence of exogenous *p*-HPA and that there are clade-specific differences in the rate of tyrosine fermentation, resulting in the modulation of *p*-cresol production (**Figure 3**). The regulation of HpdBCA decarboxylase is indirectly controlled by the global regulator, CodY, which is known to respond to nutrient availability (for a summary, see **Figure 8**).

### *p*-HPA Adversely Affected the Growth of *C. difficile* and Induced Sporulation

**Figure 4:**
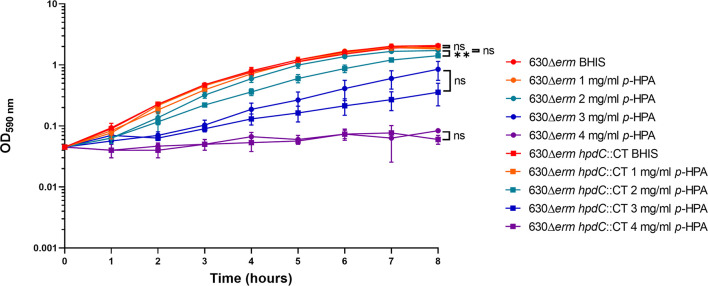
Growth analysis of 630Δ*erm* and 630Δ*erm hpdC*::CT in the presence of *p*-HPA. Growth curves were undertaken in BHIS media supplemented with 0, 1, 2, 3, and 4 mg/ml *p*-HPA over an 8-h time course. The *C. difficile* strains 630Δ*erm* (circles) and the *p*-cresol null mutant *hpdC*::CT (squares) were compared. Data represents the mean and standard deviation of three independent replicates. Statistical analysis was carried out by ANOVA, and significant differences are indicated: **p < 0.01, ns, non significant.

**Figure 5:**
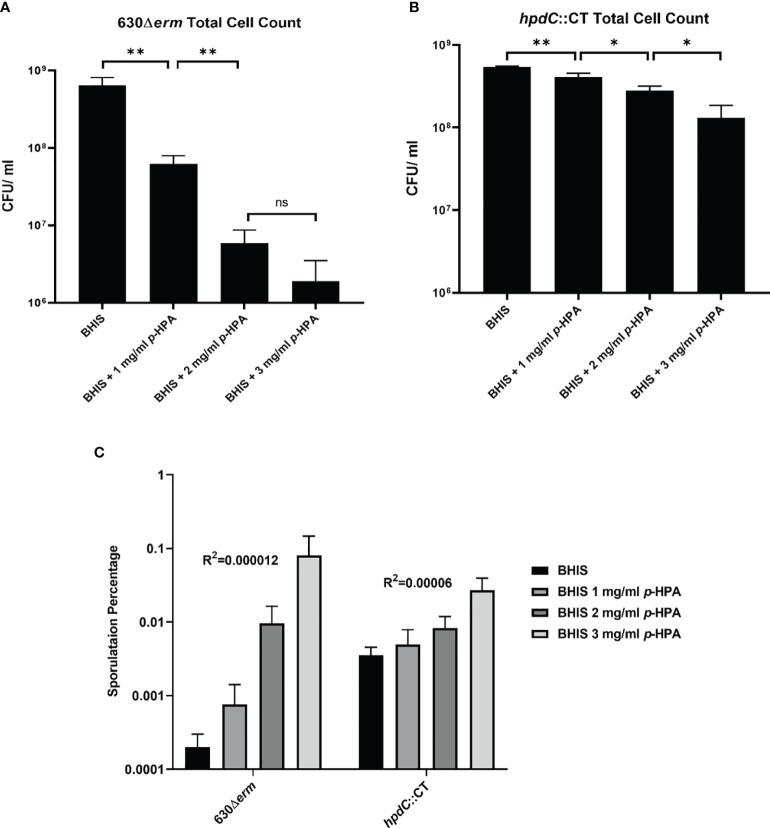
Effect of *p*-HPA and *p*-cresol on sporulation rate and total cell count. **(A)** 630Δ*erm* and **(B)** *hpdC*::CT were grown for 24 h in BHIS in the presence of 0, 1, 2, or 3 mg/ml *p*-HPA. Colony forming units (cfu) were performed per ml of culture for total cell counts and spore count (spores obtained by heating the culture at 65°C for 20 min) by plating on to BHIS plates containing 0.1% sodium taurocholate. **(C)** Percentage sporulation in 630Δ*erm* and *hpdC*::CT mutant in BHIS media alone, or media supplemented with 1, 2 or 3 mg/ml p-HPA. Statistical analysis for a correlation between *p*-HPA concentration and sporulation rate was carried out by Spearman one-tailed rank–order correlation. Statistical analysis to determine differences in total cell count was carried out by regression. Data represents the minimum of three independent replicates, error bars represent standard deviation, and significant differences are indicated: *p < 0.05, **p < 0.01, ns, non significant.

Given we have shown that *C. difficile* is able to produce *p*-cresol from two interrelated pathways, (i) tyrosine metabolism and (ii) uptake and utilization of exogeneous *p*-HPA, we set out to determine if there is a selective pressure to undertake this metabolite production. *C. difficile* strain 630Δ*erm* and a 630Δ*erm hpdC* inactivation mutant (*hpdC*::CT) were grown in 0, 1, 2, 3, and 4 mg/ml *p*-HPA and monitored over an 8 h time course (**Figure 4**). A significant growth defect was observed at ≥2 mg/ml *p*-HPA in both wild type and *hpdC*::CT mutant, showing that *p*-HPA is deleterious to *C. difficile* (**Figure 4**). Furthermore, in the presence of 2 mg/ml *p*-HPA, the growth of the *hpdC* mutant is significantly decreased compared to its wild-type counterpart (*p* < 0.01) (**Figure 4**), showing that the efficient turnover of *p*-HPA to *p*-cresol enhances the growth of *C. difficile* over the 8 h time course. The growth defect observed in the presence of *p*-HPA correlated with a significant decrease in total viable counts (vegetative cells and spores) in both the wild-type 630Δ*erm* (**Figure 5A**) and the *p*-cresol deficient mutant (**Figure 5B**), albeit more pronounced in the wild-type strain in response to increasing concentrations of *p*-HPA. The analysis of growth in the presence of *p*-HPA as a percentage of the BHIS-only control showed that, at 1, 2, and 3 mg/ml *p*-HPA, growth was reduced by 90.35 (± 1.52), 99.06 (± 0.41), and 99.71% (± 0.22), respectively, in the wild type compared to 24.61 (± 6.53), 48.43 (± 5.17), and 75.97% (± 7.71) in the *hpdC*::CT strain. Alongside this drop in total viable counts, we observed a significant increase in sporulation frequency, with a positive correlation between *p*-HPA concentration and sporulation rate (**Figure 5C**) and with a stronger correlation in the wild type (*R* ^2^ = 0.9193, *p* = 0.000012) than the *hpdC*::CT strain (*R* ^2^ = 0.8868, *p* = 0.00006), which suggests that both *p*-HPA and *p*-cresol induce sporulation in *C. difficile*.

### *p*-HPA Adversely Affects the Growth of Representative Gram-Negative Gut Bacteria

**Figure 6:**
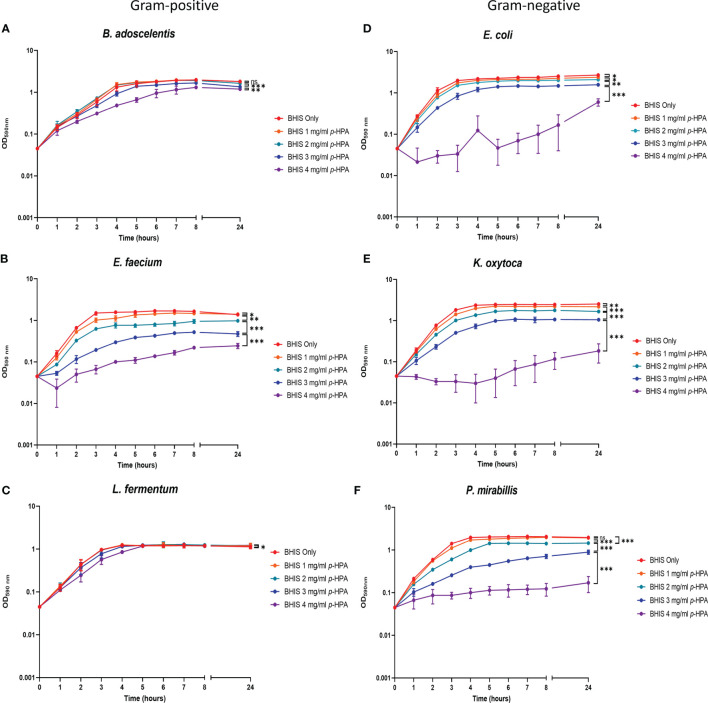
Analysis of the growth of *C. difficile* and gut commensal species in the presence of *p*-HPA. Six representative gut commensal species,three Gram-positive: **(A)** *Bifidobacterium adoscelentis*, **(B)** *Enterococcus faecium*, **(C)** *Lactobacillus fermentum*, and three Gram-negative; **(D)** *Eschericia coli*, **(E)** *Klebsiella oxytoca*, **(F)** *Proteus mirabilis* strains were grown in BHIS media with 0, 1, 2, 3, and 4 mg/ml *p*-HPA. Growth curves represent three biological replicates. ANOVA was used to determine significant differences between growth curves. Error bars represent standard deviation, and significant differences are indicated: *p < 0.05, **p < 0.01, ***p < 0.001, ns, non significant .

In addition to inhibiting the growth of *C. difficile* (**Figures 4** and **5**), *p*-HPA also inhibits the growth of commensal gut bacteria, with a reduced growth rate in the presence of *p*-HPA compared to their growth rate in media without *p*-HPA supplementation (**Figure 6**). This reduced growth rate of the Gram-negative species in the presence of increasing *p*-HPA concentrations was more pronounced than the effects on Gram-positive species (**Figure 6**). The growth of the Gammaproteobacteria *E. coli* and *K. oxytoca* was significantly inhibited by *p*-HPA (≥1 mg/ml), while *P. mirabillis* was significantly inhibited at ≥2 mg/ml (**Figure 6**). In contrast, increasing *p*-HPA concentrations had little effect on the growth rate of the Gram-positive species *B. adoscelentis* and *L. fermentum*, compared to their respective control media (**Figure 6**). The growth of *L. fermentum* was only significantly inhibited at 4 mg/ml, and *B. adoscelentis* only exhibited a growth defect at *p*-HPA concentrations of ≥3mg/ml (**Figure 6**). Surprisingly, *E. faecium* was significantly more sensitive to *p*-HPA than the other Gram-positive bacteria, exhibiting a significant growth defect at 1 mg/ml *p*-HPA (**Figure 6**) compared to its untreated control. Unlike the other Gram-positive bacteria, *C. difficile* was unable to grow in *p*-HPA ≥4 mg/ml (**Figure 4**), showing that exogenous *p*-HPA is more deleterious to *C. difficile* growth than other Gram-positive bacteria (**Figure 6**). The growth curves demonstrate that, relative to the growth rate of their untreated control, the Gram-negative species are more sensitive to inhibition than the Gram-positive species.

### *p*-HPA Affects the Cell Membrane Permeability of *C. difficile*

**Figure 7:**
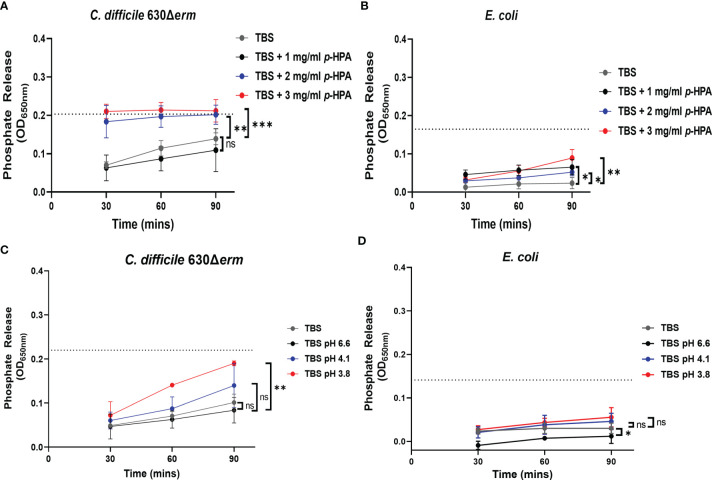
Assessing the effect of *p*-HPA compared to pH on the membrane integrity of *C. difficile* compared to *E. coli.* **(A, B)** Phosphate release assays were carried out in Tris-buffered saline (TBS) with 0, 1, 2, and 3 mg/ml *p*-HPA in *C. difficile* and *E. coli*, respectively. **(C, D)** TBS was acidified to pH 6.6, 4.1, and 3.8, respectively, with the addition of hydrochloric acid, to match the pH of the phosphate release assays undertaken in the presence of *p*-HPA. Statistical analysis was undertaken using ANOVA to compare phosphate release in *p*-HPA or acidified TBS to the TBS control. Data represents the mean and standard deviation of three independent replicates. Significant differences are indicated: *p < 0.05, **p < 0.01, ***p < 0.001, ns, non significant.

We have shown previously that *p*-cresol has a deleterious effect on the membrane integrity of Gram-negative bacteria. Here we show that, similarly to *p-*cresol, *p-*HPA has a negative impact on bacterial growth; therefore, we hypothesized that both *p*-cresol and *p*-HPA may share a similar mechanism of toxicity, *i*.*e*., disruption of the cell envelope. To determine if this was the case, we carried out phosphate release assays, as previously described with *p*-cresol (Passmore et al., 2018), with measurements every 30 min up to 90 min of exposure to *p*-HPA. We compared phosphate release in *C. difficile* strain 630Δ*erm* and *E. coli* at 0, 1, 2, and 3 mg/ml *p*-HPA to the maximum phosphate release. We found significant increases in phosphate release at 2 mg/ml (*p* = 0.0013) for *C. difficile* (**Figure 7A**) and at 1 mg/ml for *E. coli* (*p* = 0.010) (**Figure 7B**). The addition of *p*-HPA to the growth media reduces the pH in a concentration-dependent manner. To investigate whether this phosphate release was due to the acidification of the media in the presence of *p*-HPA, growth curves (**Supplementary Figures S3**, **S5**) and phosphate release assays (**Figure 7**) were undertaken in control media (0 mg/ml *p*-HPA) with the pH matched to that observed in TBS buffer in the presence of *p*-HPA, *i*.*e*., pH levels 6.6, 4.1, and 3.8 were equivalent to that for 1, 2, and 3 mg/ml *p*-HPA, respectively (**Figures 7C, D**). In *C. difficile*, we observed a significant increase in phosphate release at pH 3.8 (*p* = 0.0027), but not at pH 4.1 (*p* = 0.3039) or pH 6.6 (*p* = 0.545) when compared to the TBS control. Furthermore, the highest percentage of maximum phosphate release for *C. difficile* at low pH was only 86.5% (± 4.0) after 90 min at pH 3.8, in contrast to >95% after just 30 min in the presence of 2 mg/ml *p*-HPA, rising to >99% at 90 min (**Figures 7A, C**). Similarly, in *E. coli*, significant phosphate release was seen at ≥1 mg/ml *p*-HPA, whereas when the pH equivalent was tested (pH 6.6), phosphate release was actually significantly reduced (*p* = 0.0184); furthermore, no significant differences were observed in release at either pH 4.1 (*p* = 0.5757) or pH 3.8 (*p* = 0.2155) when compared to the control (**Figures 7B, D**).

The effect of reduced pH on growth was assessed in *C. difficile* (**Supplementary Figure S3**), which was found to be able to buffer small pH changes (to a maximum of 0.52 ± 0.08), but not return the growth media to neutral pH, which corresponds to unsupplemented media (no *p*-HPA) (**Supplementary Figure S4**) and the representative gut commensal species (**Supplementary Figure S5**). Most strains, with the exception of *L. fermentum*, had a slight growth defect in acidic media; however, the effect of acid pH (**Supplementary Figures S3** and **S4**) was not as dramatic as the effect of growth in the presence of *p*-HPA. After 8 h, 2 mg/ml *p*-HPA had a significant negative impact on the growth of *C. difficile* (*p* = 0.0064), *K. oxytoca* (*p* = 0.0073), *P. mirabillis* (*p* = 0.0073), and *E. faecium* (*p* = 0.0086), compared to growth in media without *p*-HPA but at a matched pH (pH 6.2). The growth of *E. coli* at 3 mg/ml *p*-HPA was significantly lower compared to growth at media without *p*-HPA, pH matched to pH 5.8 (*p* < 0.001) (**Supplementary Figure S4**). However, the growth rate of both *B. adoscelentis* and *L. fermentum* was similarly unaffected in both media containing *p*-HPA and media with a matched pH, compared to the *p*-HPA.

Therefore, these assays have shown that *p*-HPA induces phosphate release from the tested strains, resulting from the disruption of the cell envelope, which is only compounded by the acidity of *p*-HPA-supplemented media at the lowest pH. This suggests that, similar to *p*-cresol, *p*-HPA affects membrane integrity.

## Discussion

**Figure 8:**
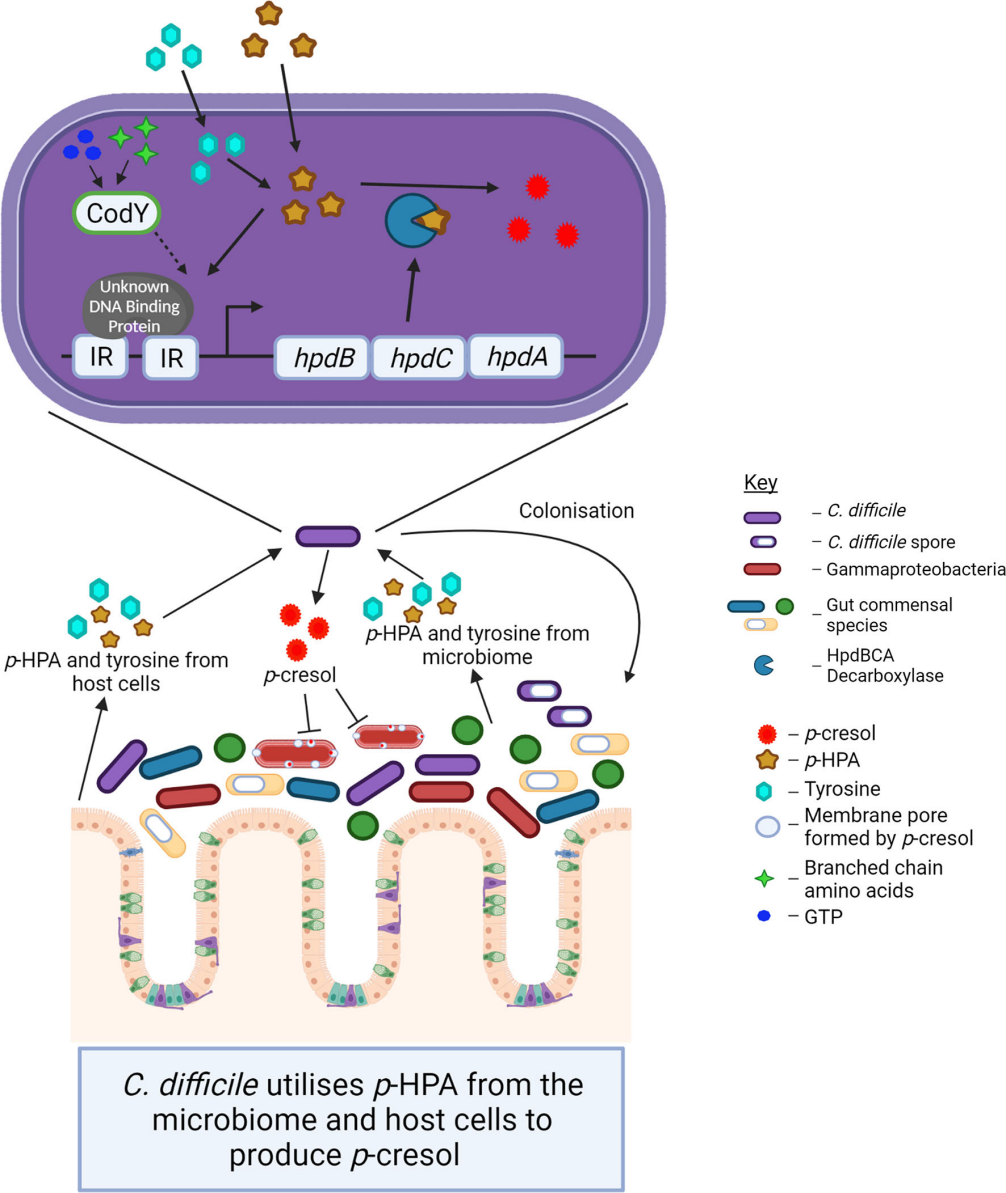
The schematic indicates the interconnected roles of the microbiome and host cells in *p*-cresol production by *C. difficile* and the impact of *p*-cresol production on the Gram-negative Gammaproteobacteria within the host microbiome.

The importance of *p*-cresol production for *C. difficile* colonization and pathogenesis combined with the recent finding that exogenous *p*-HPA induces the expression of the *hpdBCA* operon highlights the potential regulatory role for *p*-HPA in the virulence of *C. difficile* through the modulation of *p*-cresol production. In this study, we sought to identify whether *p*-cresol production in *C. difficile* from two interrelated pathways, (i) tyrosine metabolism and (ii) uptake and utilization of exogeneous *p*-HPA, is conserved throughout all the *C. difficile* lineages and to assess the effect that exogenous *p*-HPA has on the viability of *C. difficile* and other representative gut bacteria present in the healthy microbiome (summarized in **Figure 8**).

The production of *p*-cresol is an important virulence attribute for *C. difficile*, providing a competitive advantage over other commensal gut bacteria to promote dysbiosis (Passmore et al., 2018); however, the importance of its precursor *p*-HPA is yet to be determined. *p*-HPA is produced by a range of bacteria found in the gut, such as *Clostridium* and *Klebsiella* species, as well as by mammalian cells (Wishart et al., 2018), which raises the possibility of a pool of accessible *p*-HPA in the gut for conversion by *C. difficile* to *p*-cresol. Here we present new evidence that *p*-HPA is inhibitory to *C. difficile* growth and, furthermore, that a *C. difficile* mutant unable to produce the HpdBCA decarboxylase that converts *p*-HPA to *p*-cresol is significantly more susceptible to growth inhibition by *p*-HPA. This shows that the benefits of producing *p*-cresol are two-fold: (i) *p*-cresol production enables *C. difficile* to outcompete other gut bacteria and (ii) *p*-cresol production facilitates the detoxification of *p*-HPA, which has a deleterious effect on *C. difficile* growth (summarized in **Figure 8**).

There appears to be a selective advantage to drive *p*-cresol production by *C. difficile*; however, we identified that there is also a link between elevated *p*-HPA and increased sporulation rates in both the wild type and HpdBCA-deficient mutant strain. We have identified that the tyrosine metabolite *p*-HPA has a deleterious effect on the growth of *C. difficile*, which results in decreased total cell counts and increased sporulation; however, there is a trade-off between the detoxification of *p*-HPA and the production of *p*-cresol. We show that elevated *p*-cresol production reduces total cell counts and concurrently increases sporulation more predominantly in wild-type *C. difficile* rather than in a *p*-cresol-deficient mutant, which indicates a lifestyle choice of long-term survival (spore formation). This high-level *p*-cresol production comes at a cost to *C. difficile* (reduced total cell count); however, the benefit to *C. difficile* of promoting dysbiosis in the gut, by selectively killing *p*-cresol-sensitive Gram-negative species, results in a conserved mechanism of actively driving the conversion of *p*-HPA to *p*-cresol. Taken together, this suggests that there is a fine balance between *p*-HPA turnover and *p*-cresol production. We have shown in a previous study that *p*-cresol is deleterious to *C. difficile* growth when produced at levels ≥9.5 mM, and here we show that *p*-HPA is inhibitory to Growth at high concentrations (2 mg/ml, 13.1 mM). Therefore, tight regulation of *p*-cresol production is advantageous to *C. difficile*. The determination of *p*-HPA availability in the gut over the course of CDI has not been assessed and would be difficult to achieve due to the invasive nature of the sample collection. However, there is evidence that *p*-HPA is present in the human colon, and 19 µM *p*-HPA was detected in human fecal samples (Jenner et al., 2005). In addition, we previously showed that a *p*-cresol null mutant was at a significant disadvantage compared to the wild type in a mouse relapse model of CDI (Passmore et al., 2018). This is strong evidence that sufficient *p*-tyrosine, *p*-HPA, or both are available for *C. difficile* to produce *p*-cresol to maintain dysbiosis, as evidenced by our observation of a concurrent fall in Gammaproteobacteria in mice exposed to the wild type compared to the *p*-cresol-null mutant (Passmore et al., 2018). We observed previously (Harrison et al., 2020), as well as in this study, that *p*-tyrosine fermentation to produce *p*-HPA and *p*-cresol is extremely inefficient *in vitro*. Therefore, this suggests that the utilization of exogenous *p*-HPA is an important source of *p*-cresol production.

*C. difficile* is a genetically diverse species, which consists of five clades (clades 1–5) (Griffiths et al., 2010; Stabler et al., 2012), and while three further cryptic clades have been identified, these are likely to be a separate species (Dingle et al., 2014; Knight et al., 2021). Variation exists both within and between clades in major virulence factors, such as toxin production (Akerlund et al., 2008; Merrigan et al., 2010), motility (Valiente et al., 2012), and sporulation (Akerlund et al., 2008). Significant examples of this include strains of clade 4 that do not have functional toxin A (Kuijper et al., 2001; Drudy et al., 2007) and clade 5 isolates which are non-motile, such as M120 (Valiente et al., 2012), yet despite this diversity in virulence attributes between clades, the *hpdBCA* operon is highly conserved among clades 1–5. Interestingly, the more genetically divergent cryptic clades also carry *hpdBCA*-like operons. The alignments of the DNA sequences of these operons to *hpdBCA* from strain 630 showed identities of 91.1, 95.0, and 92.2% for cryptic clades C-I, C-II, and C-III, respectively (**Supplementary Figure S6**). Therefore, our finding that strains representative of clades 1–5 all show a high-level induction of the *hpdBCA* operon and high levels of *p*-cresol production in the presence of *p*-HPA suggests that this response is very well conserved and therefore of importance to *C. difficile* and, by extension, possibly CDI pathogenesis. Furthermore, given that the conversion of exogenous *p*-HPA to *p*-cresol was consistent among all representatives of clades 1–5, these findings imply that these lineages have similar capacities to transport *p*-HPA into the cell and convert it to *p*-cresol.

*p*-cresol can be produced via two pathways: firstly, from the metabolism of *p*-tyrosine and, secondly, from the uptake and decarboxylation of exogenous *p*-HPA. We identified that strains CD305 (RT023) and M120 (RT078) were the most efficient producers of *p*-HPA from *p*-tyrosine fermentation, whereas strain M68 (RT017) was the least efficient. There was a direct correlation between the ability to produce *p*-HPA from *p*-tyrosine fermentation and the subsequent conversion to *p*-cresol, which indicates strain-specific differences in the proficiency of *p*-tyrosine utilization. Interestingly, these higher *p*-cresol-producing strains, RT023 and RT078, are among the most prevalent ribotypes identified in the UK (PHE, 2019). It is noteworthy that the fermentation of *p*-tyrosine to *p*-HPA under these conditions is inefficient, which limits the ability to produce *p*-cresol. The level of *p*-cresol produced from *p*-tyrosine fermentation in the conditions tested is a minimum of 72.6-fold lower after 8 h compared to *p*-cresol produced from exogenous *p*-HPA. While these differences provide evidence for variation in *p*-tyrosine fermentation *in vitro*, the *in vivo* utilization of *p*-tyrosine may differ depending on the local availability of nutrients. In support of this, we have previously shown that environmental conditions affect *p*-cresol production *in vitro*, where we observed that production is significantly lower in rich media (BHIS) than less rich media (yeast peptone) (Dawson et al., 2011). This further suggests that exogenous *p*-HPA is a major source of *p*-cresol production.

When looking at the impact of *p*-cresol and *p*-HPA on representative strains of gut species, we found that *p*-HPA had a similar effect to our published data on the effect of *p*-cresol (Passmore et al., 2018), with both compounds being generally more toxic to Gram-negative species than Gram-positive ones. Both *K. oxytoca* and *E. coli* were significantly inhibited by 1 mg/ml (6.6 mM) *p*-HPA, whereas most Gram-positive bacteria were highly resistant to both *p*-HPA and *p*-cresol, with the exception of *E. faecium* that was significantly inhibited at this concentration. Interestingly, *p*-HPA is acidic; therefore, we sought to determine whether the effect of *p*-HPA on the growth of *C. difficile* and the Gram-negative gut bacteria was a result of the acidification of the environment by *p*-HPA. Further analysis by testing acidic pH levels via growth curves, as well as utilization of a phosphate release assay, provided evidence that the mechanism of toxicity of *p*-HPA is not limited to its acidification of the environment, as in addition, *p*-HPA also disrupts cell envelope integrity, a further similarity to *p*-cresol (Passmore et al., 2018).

The mechanisms controlling *p*-cresol production by *C. difficile* centers around the transcriptional regulation of the *hpdBCA* operon in response to *p*-HPA. We identified that *p*-HPA activates regulatory factors to initiate transcription. Here we provide evidence of the first such regulator to be involved in this process: the global regulator CodY. The deletion of *codY* renders *C. difficile* less able to convert *p*-HPA to *p*-cresol as a result of the reduced expression of the *hpdBCA* operon (**Figure 8**). This is likely to be an indirect effect as no CodY binding site has been identified directly upstream of the *hpdBCA* operon (Dineen et al., 2010). However, CodY is a global regulator with over 350 binding sites identified through the genome, 37 of which are located near to regulatory genes (Dineen et al., 2010), one of which could be the factor directly responsible for the high level of induction of the *hpdBCA* operon. Alternatively, the deletion of CodY may have led to an impaired ability for the bacteria to take up *p*-HPA. Steglich et al. have shown that R20291 CDR20291_805 and _806 are part of an ABC transporter which imports tyrosine (Steglich et al., 2018); this transporter is controlled by CodY (Dineen et al., 2010). However, they did not assess the ability of this ABC transporter system to uptake *p*-HPA. Therefore, we could speculate that the tyrosine import system may also import *p*-HPA.

Importantly, we have identified that every cell in a given population responded strongly to the presence of exogenous *p*-HPA via the transcription and translation of a SNAP-tagged HpdB subunit. However, *p*-tyrosine fermentation by *C. difficile in vitro* produced insufficient *p*-HPA to observe the induction of HpdB by SNAP tag visualization. A mutant deficient in the HpdC subunit of the HpdBCA decarboxylase complex provided evidence that all three subunits of HpdBCA are responsible for facilitating the trafficking of the enzyme throughout the cell. Given that every cell expressed HpdBCA in response to exogenous *p*-HPA, this clearly demonstrates that *p*-cresol production is not a virulence factor expressed heterogeneously within a population, such as pneumolysin in *Streptococcus pneumoniae* (Surve et al., 2018) or type three secretion systems in *Salmonella typhimurium* (Sturm et al., 2011). This indicates instead that the response to *p*-HPA is ubiquitous throughout a population as well as conserved within the species. This may suggest that the conversion of *p*-HPA to *p*-cresol by *C. difficile* is important to the survival of an individual cell as well as the wider population.

In conclusion, our results provide new insights into the impact of *p*-HPA on the viability of *C. difficile* and other bacterial species in the gut microbiome (**Figure 8**). We found that, in addition to *C. difficile* benefiting from *p*-HPA induction of *p*-cresol contributing to dysbiosis, it also benefits from the efficient removal of *p*-HPA from the immediate environment, as *p*-HPA is deleterious to *C. difficile* growth. Our findings underscore the importance of the response to *p*-HPA by demonstrating that every cell exposed to *p-*HPA responds with a high level of induction of HpdBCA decarboxylase, which is conserved in the *C. difficile* species. Rationally designed inhibitors of HpdBCA could be both highly specific and an effective target to reduce problematic *C. difficile.*

## Data Availability Statement

The mass spectrometry proteomics data have been deposited to the ProteomeXchange Consortium via the PRIDE partner repository with the dataset identifier PXD028948.

## Author Contributions

LD and MH conceived and designed the study. MH and HK carried out the experimental work and data analysis. MH and LD wrote the manuscript with contributions from BW and HK. All authors contributed to the article and approved the submitted version.

## Funding

Funding for MH was provided by the Medical Research Council (LSHTM studentship MR/N013638/1). Funding for BW was provided by the Medical Research Council (grant: MR/K000551/1). Funding for LD was provided by ISSF fellowship from the Wellcome Trust (105609/Z/14/Z; https://wellcome.ac.uk/funding) and by Athena Swan Career Restart Fellowship (from London School of Hygiene and Tropical Medicine).

## Conflict of Interest

The authors declare that the research was conducted in the absence of any commercial or financial relationships that could be construed as a potential conflict of interest.

## Publisher’s Note

All claims expressed in this article are solely those of the authors and do not necessarily represent those of their affiliated organizations, or those of the publisher, the editors and the reviewers. Any product that may be evaluated in this article, or claim that may be made by its manufacturer, is not guaranteed or endorsed by the publisher.

## Supplementary Material

Supplementary Figure S1 Confirmation of HpdB–SNAP fusion. Samples were grown in the presence and absence of 2 mg/ml *p*-HPA before undergoing western blot analysis. The indicated band was approximately the right size for the HpdB–SNAP fusion (121 kDa). The band was excised from a duplicate gel, and the presence of the HpdB–SNAP fusion was confirmed by mass spectrometry.

Supplementary Figure S2
*p*-Cresol production from 630Δ*erm* and 630Δ*erm ΔcodY*. Each strain was grown in defined media for 8 h. Samples were taken after 4 and 8 h and were analyzed by HPLC for *p*-cresol concentration. *p*-Cresol concentration was normalized to growth (as measured by OD_590nm_) at the time the sample was taken. Regression analysis was used to determine significant differences between strains in *p*-cresol production.

Supplementary Figure S3 Growth of *C. difficile* in pH matched to the presence of *p*-HPA. *C. difficile* strain was grown in BHIS alongside BHIS, with the pH lowered to 6.6, 6.2, 5.8, and 5.4 to match the pH found at 1, 2, 3, and 4 mg/ml *p*-HPA, respectively. Statistical analysis by ANOVA was used to determine differences in growth at different pH levels. Error bars represent standard deviation, and data represents the minimum of three independent replicates. Significant differences are indicated: **p* < 0.05, ***p* < 0.01, ****p* < 0.001.

Supplementary Figure S4 Growth of *C. difficile* in acidic BHIS media. 630Δ*erm* (black bars) and 630Δ*erm hpdC*::CT (gray bars) strains were grown in BHIS alongside BHIS, with the pH lowered to 6.6, 6.2, 5.8, and 5.4 to match the pH found at 1, 2, 3, and 4 mg/ml *p*-HPA, respectively, with the final pH measured after 24 h of growth. Regression analysis was used to determine whether there were any significant differences in the final pH between the two strains at each starting pH. Error bars represent standard deviation, and data represents the minimum of three independent replicates. ns, non-significant.

Supplementary Figure S5 Growth of gut commensals in pH matched to the presence of *p*-HPA. Each strain was grown in BHIS alongside BHIS, with the pH lowered to 6.6, 6.2, 5.8, and 5.4 to match the pH found at 1, 2, 3, and 4 mg/ml *p*-HPA, respectively. Representative Gram-positive **(A**-**C)** and representative Gram-negative **(D**- **F)** gut bacteria were assessed. Statistical analysis by ANOVA was used to determine differences in growth at different pH levels. Error bars represent standard deviation, and data represents the minimum of three independent replicates, where no significant difference was observed (indicated with ns).

Supplementary Figure S6 Alignment of *hpdBCA*-like operons carried by cryptic clades C-I, C-II, and C-III to *hpdBCA* operon from 630. Alignments were carried out using the Emboss Needle software from EMBL-EBI.

Supplementary Table S1 Peptides identified by mass spectrometry of HpdB–SNAP-tag. An anti-SNAP-tag western blot was run to probe the presence of HpdB fused to the SNAP-tag. Mass spectrometry was used to confirm the identity of the tested protein. Analysis was carried out in Scaffold, v5.0.1, searching Uniprot’s All Taxonomy database. Peptides identified as HpdB are listed. The peptides provide 5.9% of the complete HpdB protein sequence.
