# Supplementary figures and images for: Production of p-cresol by Decarboxylation of p-HPA by All Five Lineages of Clostridioides difficile Provides a Growth Advantage
Source: Front Cell Infect Microbiol. 2021 Oct 29;11:757599. doi: 10.3389/fcimb.2021.757599 (PMC8588808; doi:10.3389/fcimb.2021.757599)

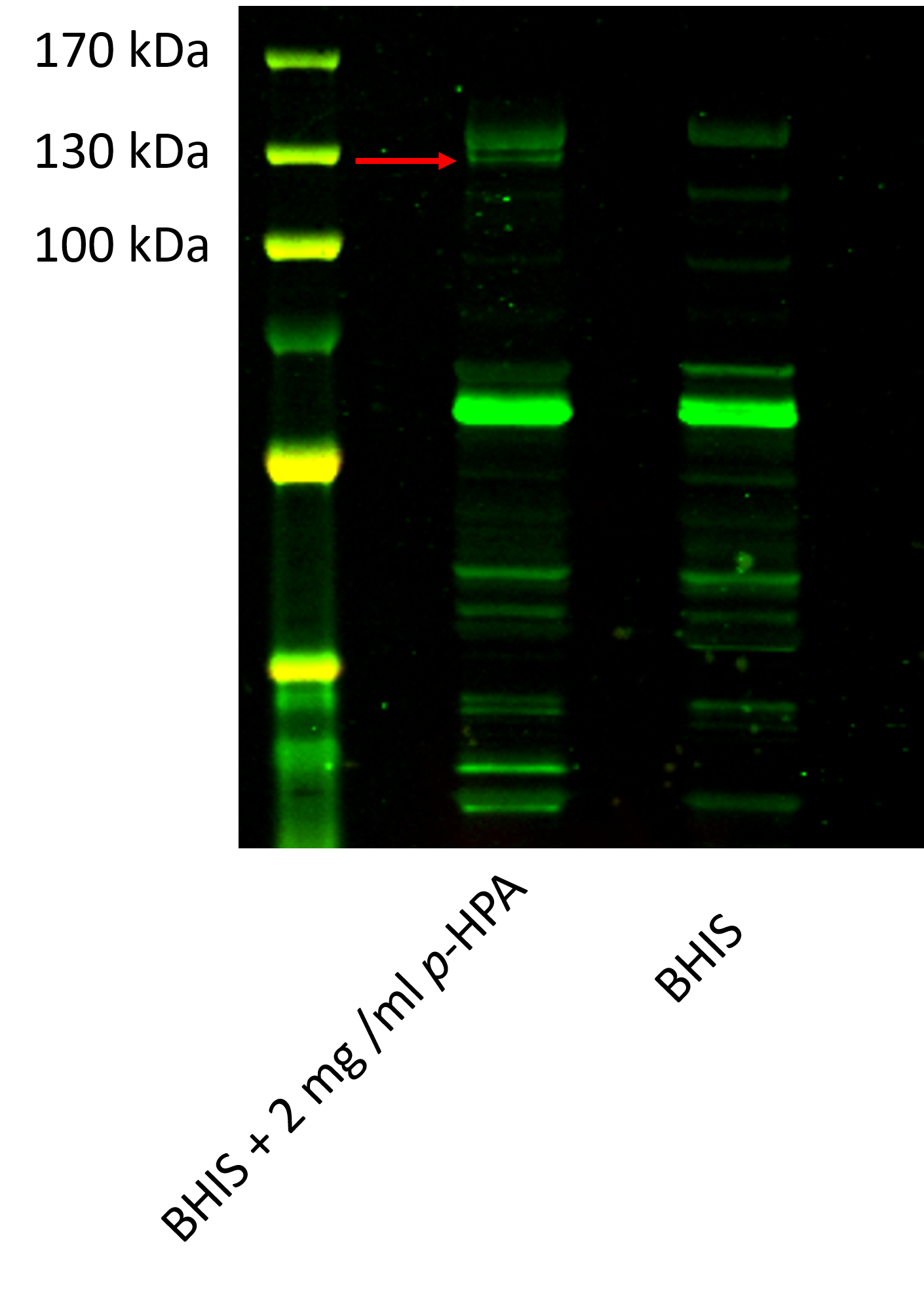

Supplement: Supplementary Figure S1 — Confirmation of HpdB–SNAP fusion. Samples were grown in the presence and absence of 2 mg/ml p-HPA before undergoing western blot analysis. The indicated band was approximately the right size for the HpdB–SNAP fusion (121 kDa). The band was excised from a duplicate gel, and the presence of the HpdB–SNAP fusion was confirmed by mass spectrometry. [file Image_1.tif]

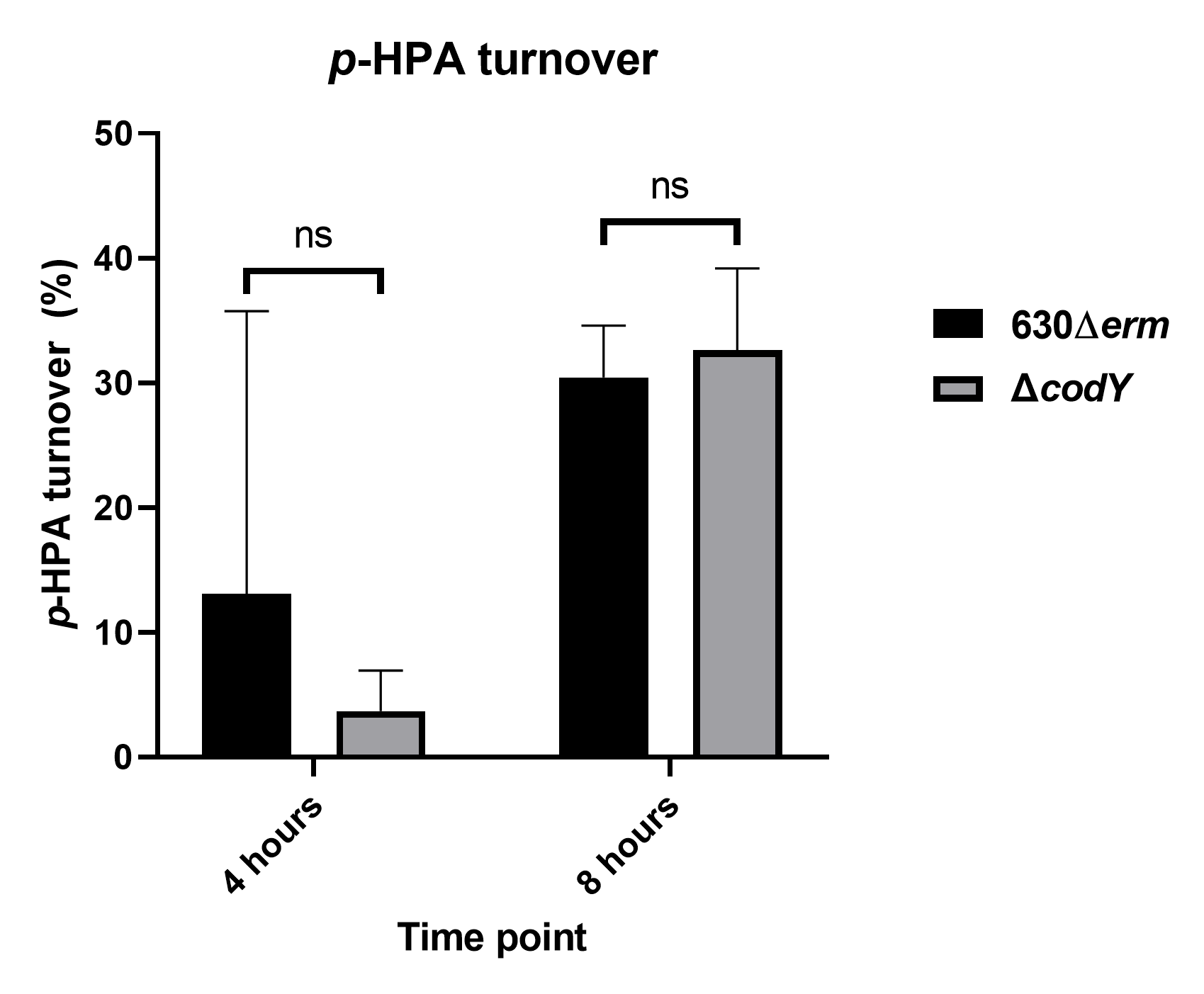

Supplement: Supplementary Figure S2 — p-Cresol production from 630Δerm and 630Δerm ΔcodY. Each strain was grown in defined media for 8 h. Samples were taken after 4 and 8 h and were analyzed by HPLC for p-cresol concentration. p-Cresol concentration was normalized to growth (as measured by OD590nm) at the time the sample was taken. Regression analysis was used to determine significant differences between strains in p-cresol production. [file Image_2.tif]

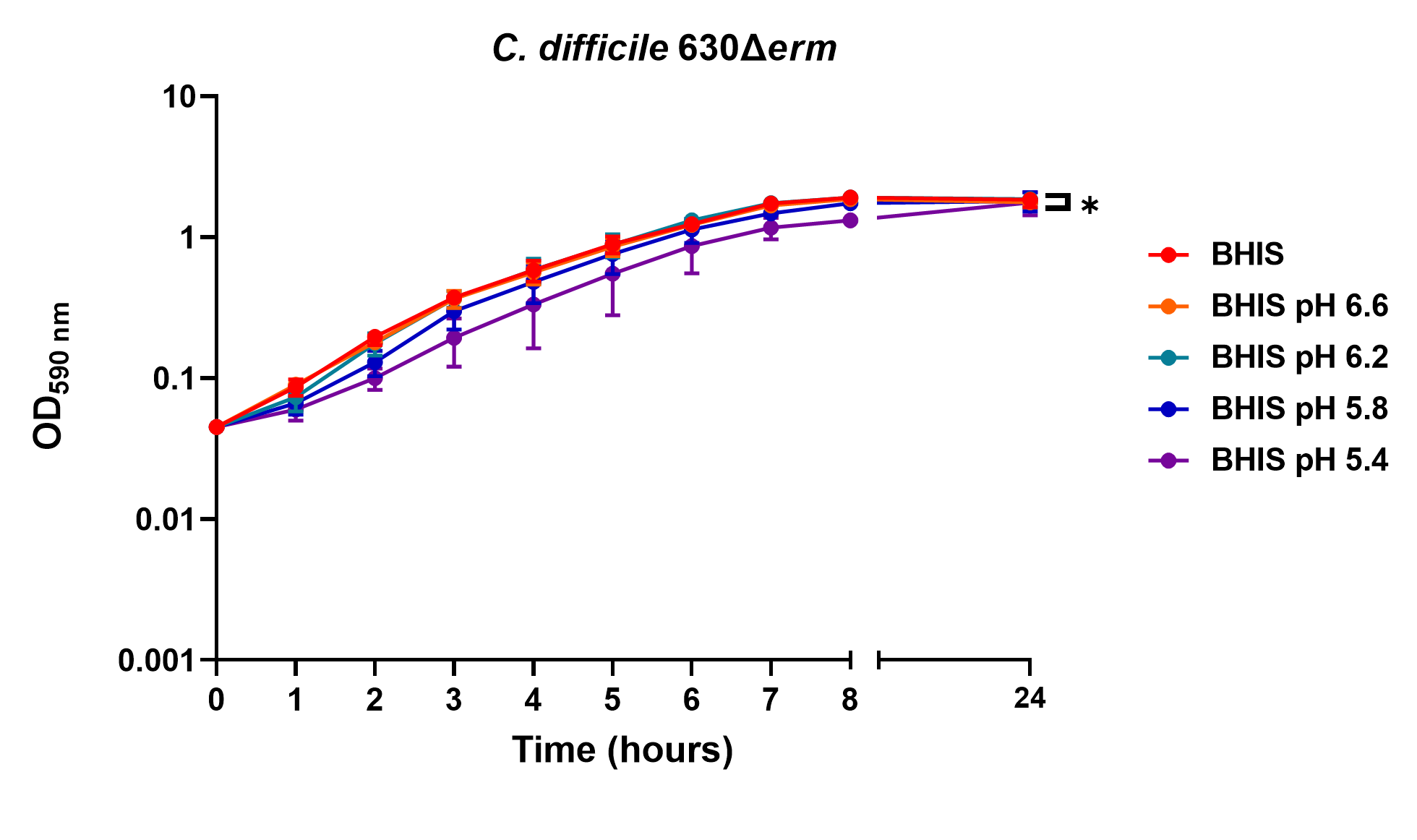

Supplement: Supplementary Figure S3 — Growth of C. difficile in pH matched to the presence of p-HPA. C. difficile strain was grown in BHIS alongside BHIS, with the pH lowered to 6.6, 6.2, 5.8, and 5.4 to match the pH found at 1, 2, 3, and 4 mg/ml p-HPA, respectively. Statistical analysis by ANOVA was used to determine differences in growth at different pH levels. Error bars represent standard deviation, and data represents the minimum of three independent replicates. Significant differences are indicated: *p < 0.05, **p < 0.01, ***p < 0.001. [file Image_3.tif]

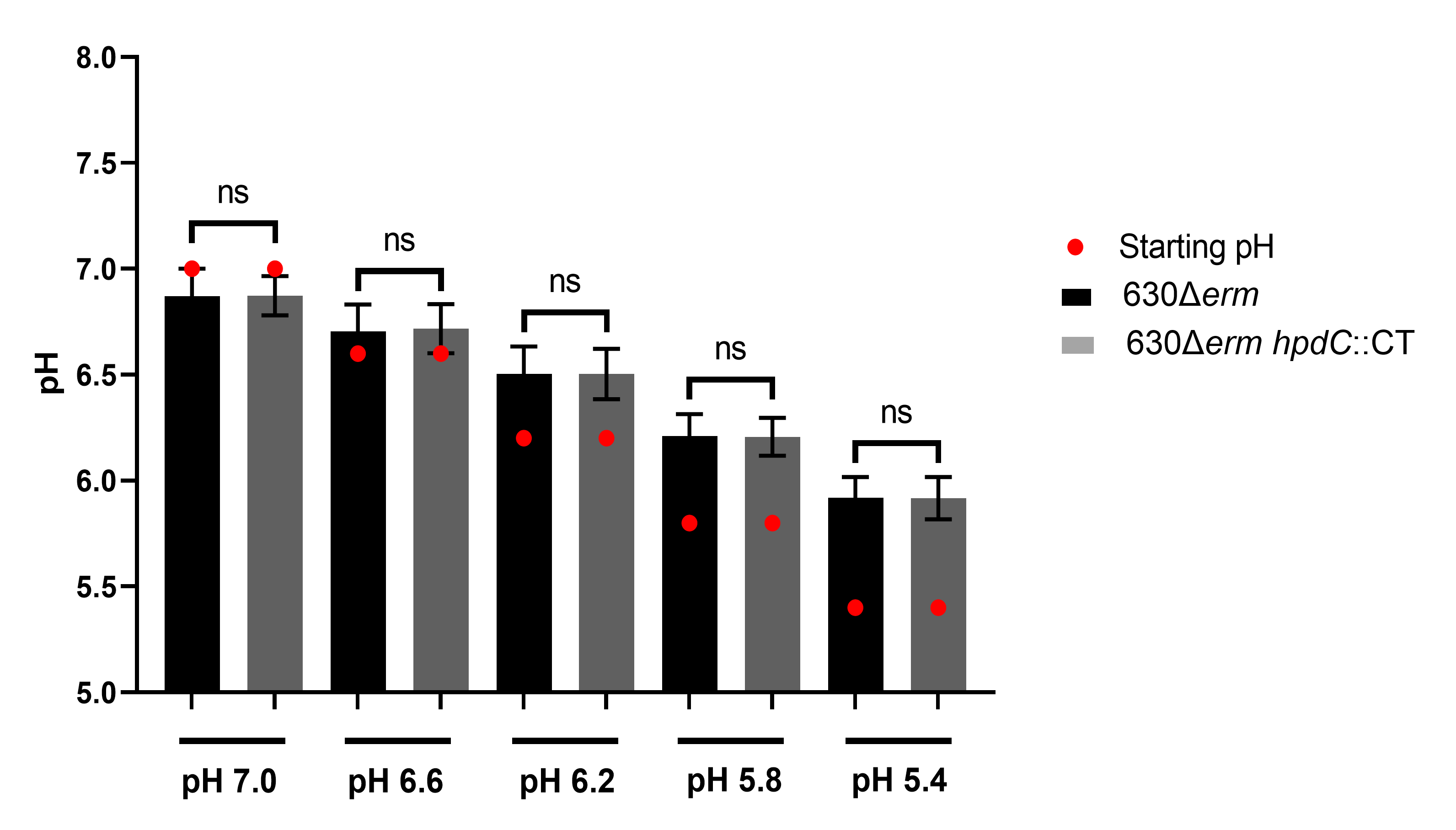

Supplement: Supplementary Figure S4 — Growth of C. difficile in acidic BHIS media. 630Δerm (black bars) and 630Δerm hpdC::CT (gray bars) strains were grown in BHIS alongside BHIS, with the pH lowered to 6.6, 6.2, 5.8, and 5.4 to match the pH found at 1, 2, 3, and 4 mg/ml p-HPA, respectively, with the final pH measured after 24 h of growth. Regression analysis was used to determine whether there were any significant differences in the final pH between the two strains at each starting pH. Error bars represent standard deviation, and data represents the minimum of three independent replicates. ns, non-significant. [file Image_4.tif]

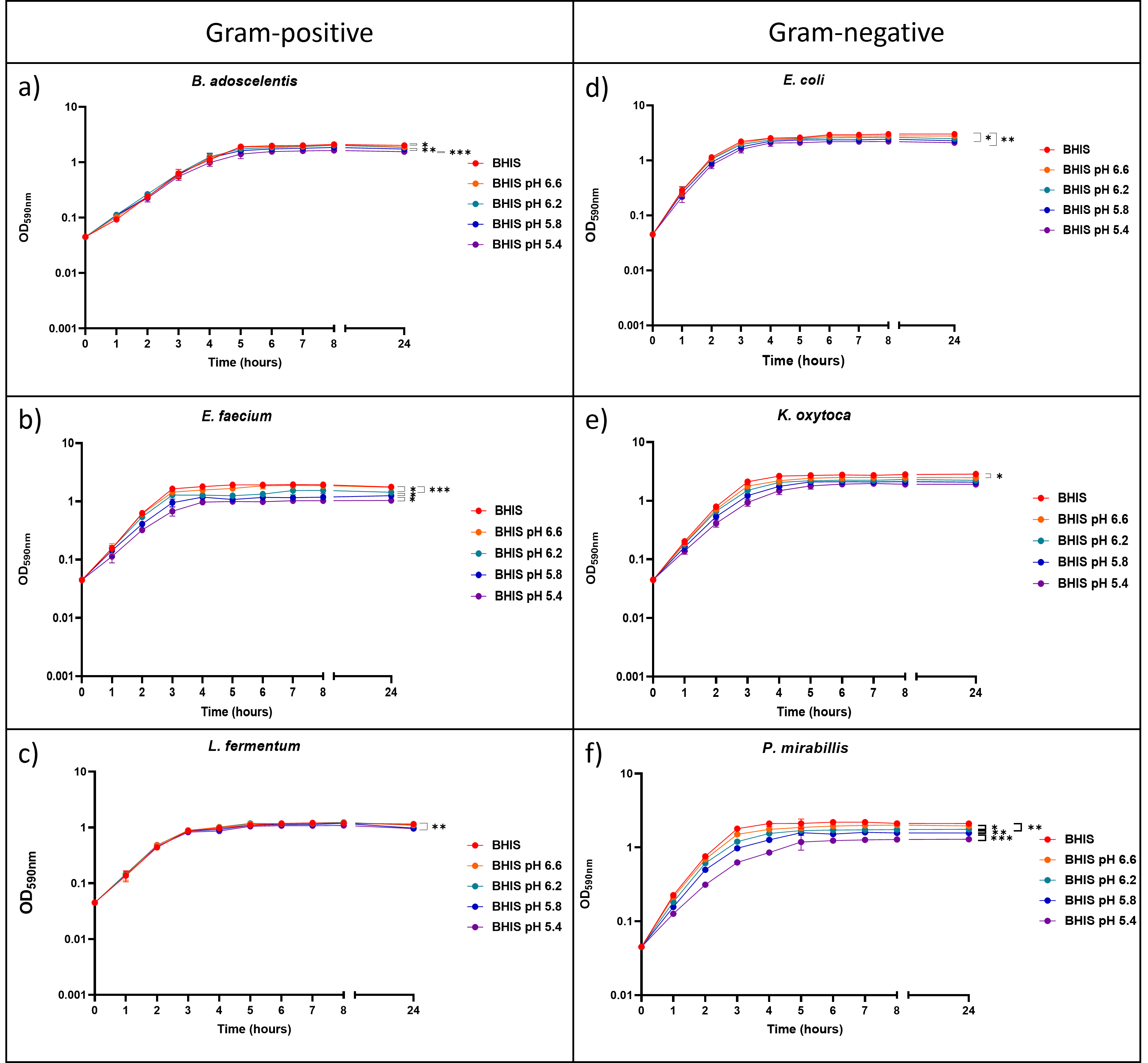

Supplement: Supplementary Figure S5 — Growth of gut commensals in pH matched to the presence of p-HPA. Each strain was grown in BHIS alongside BHIS, with the pH lowered to 6.6, 6.2, 5.8, and 5.4 to match the pH found at 1, 2, 3, and 4 mg/ml p-HPA, respectively. Representative Gram-positive (A-C) and representative Gram-negative (D- F) gut bacteria were assessed. Statistical analysis by ANOVA was used to determine differences in growth at different pH levels. Error bars represent standard deviation, and data represents the minimum of three independent replicates, where no significant difference was observed (indicated with ns). [file Image_5.tif]

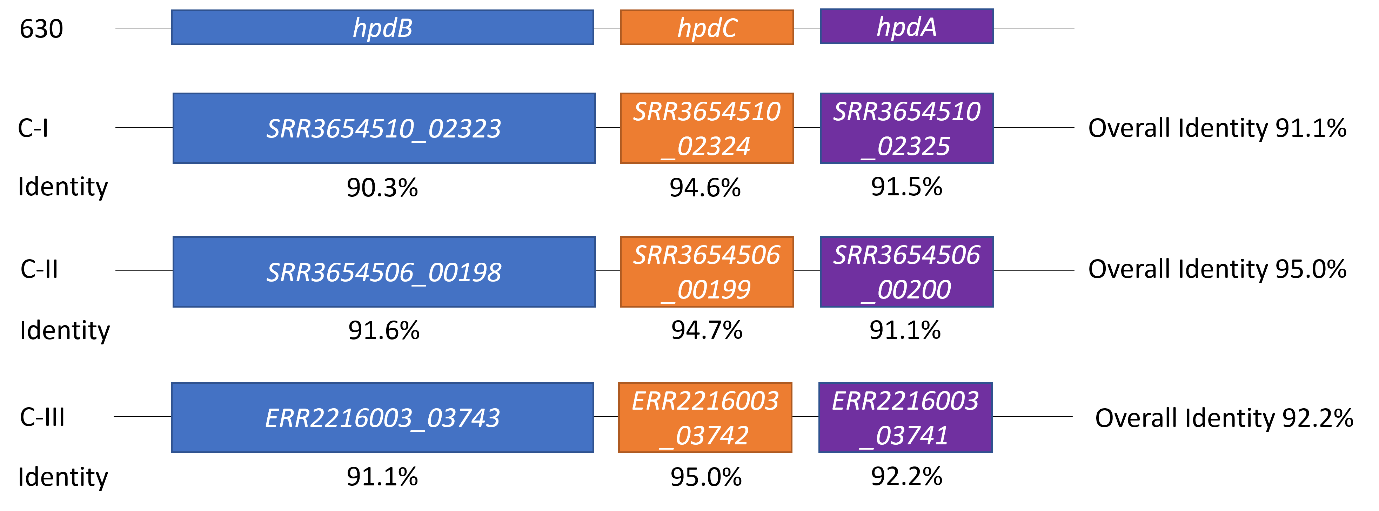

Supplement: Supplementary Figure S6 — Alignment of hpdBCA-like operons carried by cryptic clades C-I, C-II, and C-III to hpdBCA operon from 630. Alignments were carried out using the Emboss Needle software from EMBL-EBI. [file Image_6.tif]
