# Supplementary material for: Production of p-cresol by Decarboxylation of p-HPA by All Five Lineages of Clostridioides difficile Provides a Growth Advantage
Source: Front Cell Infect Microbiol. 2021 Oct 29;11:757599. doi: 10.3389/fcimb.2021.757599 (PMC8588808; doi:10.3389/fcimb.2021.757599)
Supplement: Supplementary Table S1 — Peptides identified by mass spectrometry of HpdB–SNAP-tag. An anti-SNAP-tag western blot was run to probe the presence of HpdB fused to the SNAP-tag. Mass spectrometry was used to confirm the identity of the tested protein. Analysis was carried out in Scaffold, v5.0.1, searching Uniprot’s All Taxonomy database. Peptides identified as HpdB are listed. The peptides provide 5.9% of the complete HpdB protein sequence. [file Table_1.docx]

| Peptide | Identity to HpdB |
| --- | --- |
| KSDGDIPVVR | 99% |
| SDGDIPVVR | 99% |
| LASNTADELTK | 100% |
| QFADEGMTVEEAR | 100% |
| QAINVLER | 100% |
| VCEEAQSLYAK | 100% |

**Supplementary table 1. Peptides identified by mass spectrometry of HpdB-SNAP-tag**. An anti-SNAP-tag western blot was run to probe identify the presence of HpdB fused to the SNAP-tag. Mass spectrometry was used to confirm the identity of the tested protein. Analysis was carried out in Scaffold v5.0.1 searching Uniprot’s All Taxonomy database. Peptides identified as HpdB are listed. The peptides provide 5.9% of the complete HpdB protein sequence.
